# Serum Uric Acid as a Sex‐Dependent Risk Marker of Post‐Stroke Epilepsy After Acute Ischemic Stroke: Complementary Mendelian Randomization and Cohort Analyses

**DOI:** 10.1002/cns.70970

**Published:** 2026-06-08

**Authors:** Yan Cheng, Ji Li, Yunzhou Yang

**Affiliations:** ^1^ Department of Neurology Lu'an People's Hospital (The Affiliated Lu'an Hospital of Anhui Medical University) Lu'an Anhui China

**Keywords:** acute ischemic stroke, causal inference, Mendelian randomization, post‐stroke epilepsy, predictive utility, restricted cubic spline, serum uric acid, sex interaction

## Abstract

**Background:**

Observational studies have consistently reported associations between serum uric acid (SUA) and post‐stroke epilepsy (PSE) in acute ischemic stroke (AIS), including a recently published large cohort study identifying a U‐shaped relationship. However, whether these associations reflect causality and whether SUA provides incremental predictive value beyond established clinical factors remains unknown.

**Methods:**

we conducted a two‐sample Mendelian randomization (MR) analysis to test whether lifelong genetically predicted serum urate shares a genetic causal architecture with general epilepsy susceptibility, using 299 independent genetic instruments. In parallel, we performed a prespecified secondary analysis of a multicenter AIS cohort (*n* = 21,459) using multivariable logistic regression with restricted cubic splines (RCS) to characterize the observational SUA–PSE relationship. Formal sex interaction testing (Wald tests) and sex‐stratified spline analyses were performed. Predictive utility was evaluated through discrimination (AUC), calibration, decision curve analysis (DCA), and internal validation with 1000 bootstrap resamples. The two analytical components address related but distinct biological questions: observational analysis tests the admission‐SUA‐to‐1‐year‐PSE pathway, while MR tests whether lifelong genetically predicted urate shares a causal architecture with general epilepsy susceptibility.

**Results:**

MR analysis found no evidence supporting a shared lifelong genetic causal architecture between serum urate and general epilepsy susceptibility across all four methods (IVW: OR = 1.043, 95% CI 0.926–1.174, *p* = 0.487; MR‐Egger *p* = 0.582; weighted median *p* = 0.589; weighted mode *p* = 0.996), with no pleiotropy (Egger intercept *p* = 0.865) or heterogeneity (Cochran Q *p* = 0.206). In the observational cohort (*n* = 21,459; 936 PSE events (4.36%)), RCS analyses confirmed a significant nonlinear SUA–PSE association (P for nonlinearity < 0.001). Importantly, a formal sex‐by‐SUA interaction revealed strikingly divergent patterns (overall *p* < 0.001; nonlinear component *p* = 0.0002): Women in the highest SUA tertile had markedly elevated PSE risk (adjusted OR = 2.33, 95% CI 1.71–3.19), a finding confirmed with sex‐specific tertile cut‐points (OR = 1.50, *p* = 0.005). The apparent protective association in men using overall tertiles (OR = 0.36, 95% CI 0.28–0.46) was attenuated and non‐significant with sex‐specific cut‐points (OR = 0.84, *p* = 0.16), indicating sensitivity to stratification method. Adding SUA to a base clinical model produced a modest but statistically significant increment in discrimination (AUC: 0.8498 to 0.8607, ΔAUC = 0.011, DeLong *p* = 0.001) with consistent positive net benefit on DCA.

**Conclusions:**

Genetically predicted lifelong SUA does not share a causal genetic architecture with general epilepsy susceptibility; this null does not exclude acute post‐stroke, context‐specific mechanisms, which operate on biological timescales outside the scope of Mendelian randomization. The sex‐dependent observational association (female high‐SUA OR = 1.50 with sex‐specific tertiles; sex × SUA continuous interaction *p* = 3.99 × 10^−9^) generates the hypothesis that sex‐stratified SUA monitoring merits prospective investigation, pending external validation.

## Graphical abstract

In 21,459 acute ischemic stroke patients, Mendelian randomization found no shared genetic causal link between serum uric acid and epilepsy. Elevated admission uric acid was a robust risk marker for post‐stroke epilepsy in women but not men, with the apparent male protective association sensitive to stratification method.

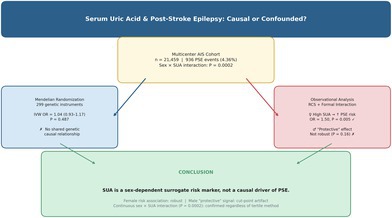

## 1 Introduction

Acute ischemic stroke (AIS) remains a leading cause of mortality and long‐term disability worldwide [1, 2]. Post‐stroke epilepsy (PSE) affects a clinically meaningful proportion of survivors and represents an important complication requiring early risk stratification [3]. Serum uric acid (SUA), the final oxidation product of purine metabolism, has attracted sustained interest as a candidate biomarker. Uric acid is a potent endogenous antioxidant accounting for a substantial proportion of plasma free radical scavenging capacity [4], and experimental studies have suggested neuroprotective effects during cerebral ischemia [5]. However, clinical evidence regarding SUA in AIS is inconsistent: Some studies reported protective associations [6, 7, 8], whereas others linked elevated SUA to increased vascular risk or poorer outcomes [9, 10, 11], and meta‐analyses have yielded conflicting conclusions [12, 13].

One potential explanation for these discrepancies is the widespread assumption of linearity. Biological systems often exhibit nonlinear behavior when a biomarker exerts both protective and deleterious effects depending on concentration [14, 15]; flexible modeling techniques such as restricted cubic splines (RCS) can capture such threshold effects [16]. Another underexplored dimension is sex‐specific heterogeneity. SUA levels differ substantially between men and women due to hormonal regulation and metabolic differences; estrogen promotes uric acid excretion, resulting in lower SUA levels in premenopausal women [17]. Sex differences in post‐stroke pathophysiology and vascular risk profiles further suggest that the SUA–PSE association may differ by sex [18], yet most prior studies adjusted for sex as a covariate rather than formally testing interaction effects [19, 20].

Recent large‐scale stroke cohorts have enabled more refined secondary analyses [21, 22, 23]. In a recently published cohort analysis using the same multicenter AIS dataset, Liu et al. reported a U‐shaped association between admission SUA and PSE, with evidence of sex‐stratified variation [24]. The present study had two related but distinct aims. First, in the observational component, we tested whether admission serum uric acid is associated with 1‐year post‐stroke epilepsy after acute ischemic stroke, and whether the association is sex‐dependent. Second, in the Mendelian randomization component, we tested whether lifelong genetically predicted serum urate shares a genetic causal architecture with general epilepsy susceptibility, as the closest available genetic proxy for the PSE phenotype. This study extends the prior analysis of the same public cohort by Liu D et al. [24], in four specific ways: (1) formal Wald testing of the sex × SUA interaction (not previously performed); (2) sex‐specific tertile sensitivity analysis demonstrating that the apparent male protective effect at high SUA is a stratification artifact; (3) Mendelian randomization against the largest cross‐ancestry urate genome‐wide association study (GWAS) to date (*N* = 1,029,323); and (4) an explicit triangulation framework for interpreting the concordant observational + null MR pattern.

## 2 Methods

### 2.1 Study Design and Data Source

This study is a prespecified secondary analysis of a publicly available multicenter retrospective cohort obtained from the Dryad Digital Repository [25]. The source dataset was released in association with the parent study by Liu et al. (eLife, 2024) [26], that developed predictive models for secondary epilepsy within one year after acute ischemic stroke (AIS). A separate recent cohort report using the same dataset examined the nonlinear association between admission serum uric acid (SUA) and post‐stroke epilepsy (PSE) [24]; the present study extends that literature by integrating Mendelian randomization with formal sex‐interaction modeling to address the distinct scientific question of whether the observed SUA–PSE association reflects causality and whether it is modified by sex.

The study adhered to the principles of the Declaration of Helsinki. This was a secondary analysis of a publicly available, de‐identified dataset obtained from the Dryad Digital Repository. No additional institutional ethics approval or informed consent was required for the present secondary analysis under local policy for analysis of publicly available anonymized data. The enrollment period in the original cohort spanned from June 2017 to June 2022, consistent with the original cohort design.

### 2.2 Study Population

**Inclusion criteria in the parent cohort included:**

- Patients aged 18–90 years at admission.
- Diagnosed with acute ischemic stroke and hospitalized for treatment.
- Exclusion criteria in the parent cohort included: History of stroke or transient ischemic attack; history of traumatic brain injury, intracranial tumors, or cerebral vascular malformations that may cause epilepsy; history of epilepsy or prior antiseizure medication use for seizure prevention or other indications; and death within 72 h after stroke onset. Patients lost to follow‐up (without outpatient records or unreachable by phone) and those who died within three months of the index stroke were also excluded during primary cohort construction.

The de‐identified dataset used for this secondary analysis contained two patients aged 17 years; therefore, the analytical age range was 17–90 years, and the final locked sample size remained 21,459. Retention of these two records was solely to preserve reproducibility with the publicly available dataset; exclusion did not materially change the results (Table S11).

To evaluate the impact of retaining two participants aged 17 years (< 0.01% of the analytic sample), we performed a sensitivity analysis restricted to age ≥ 18 years (*n* = 21,457); all primary and secondary estimates were materially unchanged (new Table S11). To assess robustness to severe‐stroke cases, we additionally repeated the primary observational analyses restricted to patients with admission National Institutes of Health Stroke Scale (NIHSS) ≤ 15 (Table S5).

### 2.3 Exposure Variable: Serum Uric Acid

Serum uric acid (SUA) was measured at admission as part of routine biochemical assessment. For the primary analysis, SUA was treated as a continuous variable. To explore potential nonlinear associations, SUA was modeled using restricted cubic splines (RCS) with four knots. Knot locations were prespecified at the 5th, 35th, 65th, and 95th percentiles of the SUA distribution, a strategy commonly recommended to balance flexibility and stability in large datasets [16]. The reference value for spline modeling was set at the median SUA level of the cohort. Overall and nonlinear components were evaluated using joint Wald χ^2^ tests within the spline framework. (Table S1). Because the upper‐tail inflection is interpreted descriptively rather than as a validated clinical cut‐point, we retained the prespecified 4‐knot specification as the reference model and did not include alternative‐knot models as formal supplementary sensitivity analyses.

Sex was coded as 0 = female and 1 = male throughout all analyses.

### 2.4 Outcome Definition

The primary outcome was post‐stroke epilepsy (PSE) within one year after the index acute ischemic stroke, as defined in the source dataset (secondary epilepsy; binary yes/no) and ascertained in the parent cohort through structured clinical documentation with outpatient records and telephone follow‐up. The present analysis used the prespecified outcome as recorded in the unified dataset; no alternative severity thresholds were applied.

The outcome was ascertained by the parent study as a 1‐year binary indicator (PSE present vs. absent within 1 year of the index stroke); time‐to‐event (date‐of‐first‐seizure) was not captured in the source dataset, precluding survival analysis or temporal stratification within the 1‐year window.

### 2.5 Statistical Analysis

Descriptive Statistics Continuous variables were expressed as mean ± standard deviation or median (interquartile range), depending on distribution. Normality was assessed using the Shapiro–Wilk test. Group comparisons between No PSE and PSE were performed using Student's *t*‐test for normally distributed variables, Mann–Whitney *U* test for non‐normally distributed variables, and χ^2^ test for categorical variables. All tests were two‐sided, and *p* < 0.05 was considered statistically significant.

### 2.6 Multivariable Logistic Regression

Multivariable logistic regression models were constructed to estimate odds ratios (ORs) and 95% confidence intervals (CIs) for the association between SUA and PSE. Three hierarchical models were built: Model 1: Crude model, Model 2: Adjusted for age and sex, Model 3: Fully adjusted model including age, sex, NIHSS score, cortical involvement (represented by both a binary indicator for any cortical lobe affected and the involved‐lobe count, allowing the model to capture both the threshold and gradient effects of lesion extent), urea, and creatinine. SUA was entered into regression models in two ways: As a linear continuous variable and as a nonlinear variable using restricted cubic splines. The prespecified model comparison and reporting framework is summarized in Table S4.

Covariate selection was prespecified and locked prior to outcome analysis to avoid overfitting of the interaction model and to preserve the validity of the integrated framework multiplicity justification (see Statistical Analysis subsection). The covariate set (age, sex, NIHSS, cortical involvement as a binary indicator, and involved‐lobe count, urea, and creatinine) was restricted to seven parsimoniously chosen clinical and laboratory variables. Variance Inflation Factors for the full covariate set are reported in Table S12 (all VIF < 5; is_cortical 4.01 and rangelobe 4.07, consistent with acceptable collinearity given that is_cortical = I(rangelobe > 0)).

### 2.7 Assessment of Nonlinearity

Nonlinearity was formally assessed using joint Wald χ^2^ tests within the rms framework, decomposing the overall association into linear and nonlinear components. A statistically significant nonlinear component (*p* < 0.05 for the nonlinear Wald χ^2^ test) was taken as evidence of a nonlinear SUA–PSE association. Adjusted spline curves were plotted with 95% confidence intervals to visualize the relationship between SUA and the predicted probability of PSE.

### 2.8 Interaction Analysis

To evaluate potential effect modification by sex, an interaction term (SUA × sex) was added to the fully adjusted model. Statistical significance of interaction was assessed using Wald tests. If interaction was significant (*p* < 0.05), sex‐stratified analyses were performed. Separate spline models were fitted in men and women to visualize heterogeneity in association patterns.

Overall‐cohort tertile cut‐points (Low < 316.4, Mid 316.4–356.1, High ≥ 356.1 μmol/L) were used as the primary sex‐stratified analysis for comparability with prior observational literature; sex‐specific tertile cut‐points were pre‐specified as a sensitivity analysis to test robustness to stratification method, included in the locked analytical pipeline (set.seed(20260221); model specifications frozen prior to outcome analysis; see Supplementary Script E).

### 2.9 Supportive Performance Analyses: Discrimination and Internal Validation

Discrimination was assessed using the area under the receiver operating characteristic curve (AUC) and compared between a base clinical model and a model additionally including SUA. Internal validation used 1000 non‐parametric bootstrap resamples drawn with replacement from the original dataset; resampling was not stratified by outcome, as the large event count (*n* = 936) ensured that all bootstrap replicates contained a sufficient number of events for model fitting (binomial approximation: 95% of resamples contained 870–1000 events). Optimism‐corrected AUC was estimated and used to assess model overfitting.

### 2.10 Supportive Performance Analyses: Calibration and Decision Curve Analysis (DCA)

Calibration was evaluated using calibration plots and the Brier score [27]. Decision curve analysis [28] was used to examine potential net benefit across a range of threshold probabilities. A modest but positive incremental net benefit after adding SUA was interpreted as supportive rather than primary evidence.

All statistical analyses were performed using R (version 4.5.2) with packages rms, pROC, boot, and rmda. Two‐sided *p* < 0.05 was considered statistically significant. All modeling procedures were prespecified and frozen prior to interpretation of outcome distributions to minimize data‐driven bias. Key analytic metrics from the locked analysis are summarized in Table S2; a reproducibility checklist is provided in Table S3. Detailed methods, analytical code availability, and Supplementary Tables and figures are provided in the Supporting Information [see Additional file 1].

All hypothesis tests reported (four MR methods, Wald tests for overall, linear, and nonlinear SUA effects, sex × SUA interaction, nonlinear‐interaction component, sex‐stratified tertile ORs, AUC comparison, and decision curve analysis) were prespecified components of a single integrated analytical framework with a priori biological hypotheses. Formal Bonferroni correction was therefore not applied; however, we note that the sex × SUA interaction (*p* = 3.99 × 10^−9^) would survive any conventional multiplicity adjustment.

### 2.11 Mendelian Randomization

To complement the observational analyses, a two‐sample Mendelian randomization (MR) was performed to test whether lifelong genetically predicted serum urate shares a genetic causal architecture with general epilepsy susceptibility. The MR component was reported in accordance with the STROBE‐MR framework [29]; a completed STROBE‐MR checklist is provided as Additional file 7. GWAS summary data were obtained from the IEU Open GWAS platform. The exposure GWAS was a large cross‐ancestry meta‐analysis of serum urate (Cho C et al., *Nat Commun* 2024;15:3441; *N* = 1,029,323; IEU ID: ieu‐b‐5137) [30]. The outcome GWAS was a large epilepsy meta‐analysis (ILAE Consortium on Complex Epilepsies, *Nat Genet* 2023;55:1471–1482; 29,944 cases/52,538 controls; IEU ID: ebi‐a‐GCST90018840) [31]. We note that general epilepsy susceptibility was used as the outcome GWAS because no PSE‐specific GWAS is currently publicly available at a consortium scale. The resulting MR inference is therefore indirect and limited to the lifelong genetic dimension of the SUA–epilepsy relationship; it does not test the acute post‐stroke clinical pathway. Genetic instruments were selected at genome‐wide significance (*p* < 5 × 10^−8^) and pruned for linkage disequilibrium (r^2^ < 0.001, 10,000 kb window), yielding 323 independent instruments. After allele harmonization with the epilepsy outcome GWAS (removing 2 incompatible single‐nucleotide polymorphisms (SNPs), 6 palindromic SNPs with ambiguous strand assignment, and 16 SNPs absent or mismatched in the outcome GWAS), 299 SNPs were retained for the primary MR analysis. Per‐SNP F‐statistics (F_i_ = β^2^_exposure/SE^2^_exposure) were computed for all 299 retained instruments. Mean F = 169.5, median 49.1, minimum 24.2, maximum 20,210; zero instruments had F < 10, indicating the absence of weak‐instrument bias (see new Table S10). The primary method was inverse variance weighted (IVW); sensitivity analyses included MR‐Egger regression, weighted median, and weighted mode. The three core MR assumptions [32] were empirically assessed: (i) relevance, via F‐statistic; (ii) independence, via LD pruning; (iii) exclusion restriction, via MR‐Egger intercept test (intercept = −0.000381, *p* = 0.865, no directional pleiotropy), Cochran's Q test (Q = 317.80, df = 298, *p* = 0.206, no significant heterogeneity), and leave‐one‐out analysis (Figure S4, no single SNP driving the estimate). A sex‐stratified two‐sample MR analysis was not feasible because sex‐stratified urate GWAS summary statistics of comparable sample size are not currently available in IEU OpenGWAS; the sex‐combined Cho et al. 2024 GWAS represents the largest available resource. The observational sex × SUA interaction (β = −0.00786, *p* = 3.99 × 10^−9^; OR per 50 μmol/L = 0.675, 95% CI 0.592–0.769) therefore serves as the primary evidence for sex heterogeneity in this study. All MR analyses were performed using Two Sample MR v0.7.0 in R 4.5.2. Full MR results and code are provided in the Supporting Information.

## 3 Results

### 3.1 Baseline Characteristics (Figure 1, Table 1)

**FIGURE 1:**
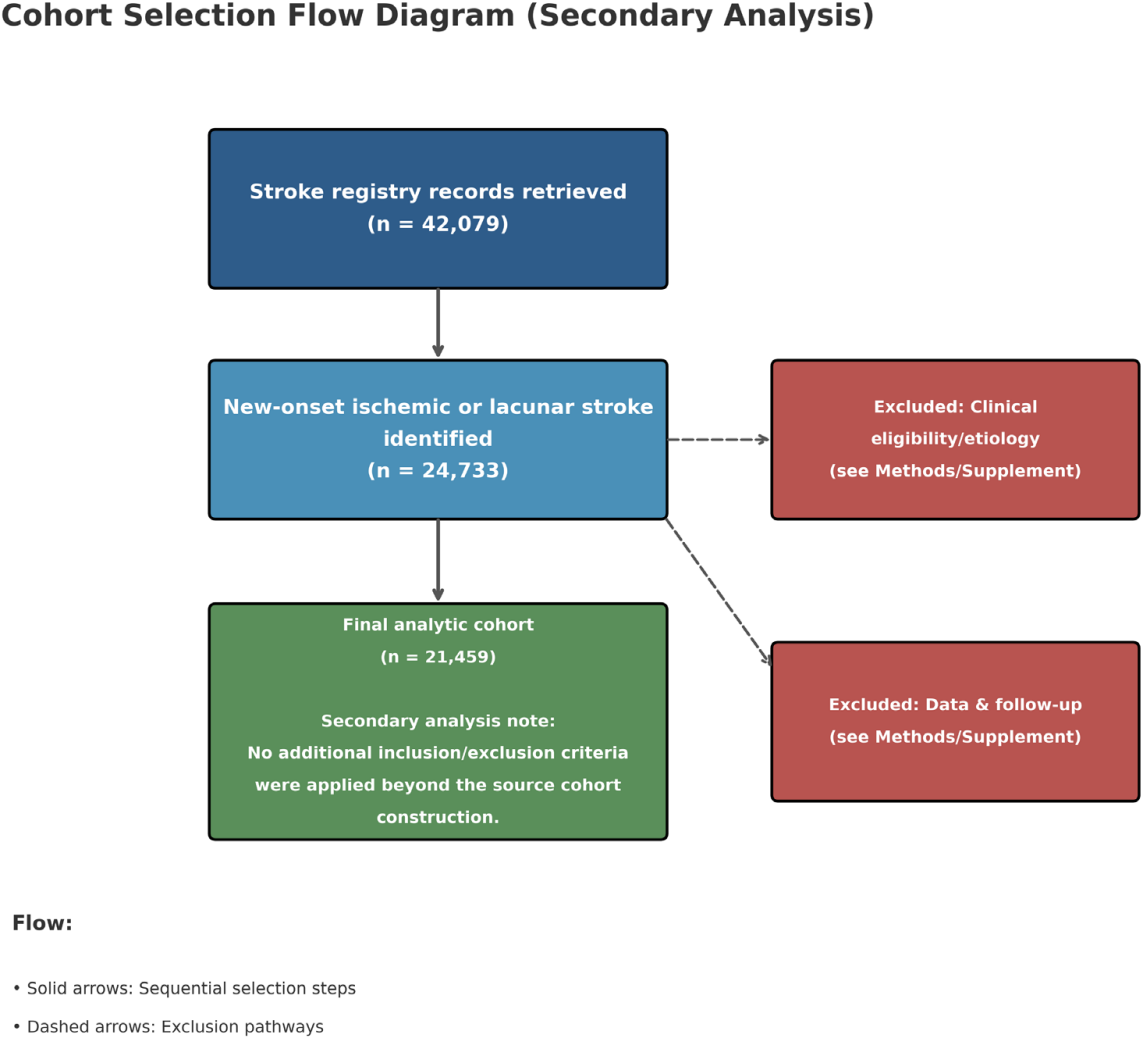
Cohort flow diagram of the study population (secondary analysis). Flowchart showing screening and exclusions from the stroke registry records to the final analytic cohort. From 42,079 stroke database records, 24,733 patients with new‐onset ischemic or lacunar stroke were identified. Exclusions included: Other seizure etiologies (brain tumors, vascular malformations, traumatic brain injury; *n* = 865), history of seizures (*n* = 152), in‐hospital death (*n* = 1,444), and loss to follow‐up or death within three months (*n* = 813), yielding a final analytic cohort of 21,459 patients. Detailed exclusion criteria are described in the parent study [26].

**TABLE 1:** Baseline characteristics (No PSE vs. PSE).

Variable	No PSE	PSE	*p*
Age (years)	66.48 ± 12.29	64.90 ± 13.45	< 0.001
Female, *n* (%)	10,252 (50.0%)	364 (38.9%)	< 0.001
NIHSS score	7.85 ± 2.86	11.50 ± 2.51	< 0.001
Cortical involvement, *n* (%)	1,208 (5.9%)	121 (12.9%)	< 0.001
Rangelobe (count, 0–5)	0.13 ± 0.58	0.28 ± 0.84	< 0.001
Admission SUA (μmol/L)	344.19 ± 58.05	343.01 ± 74.22	0.632
Urea (mmol/L)	6.41 ± 1.44	6.32 ± 1.28	0.040
Creatinine (μmol/L)	84.93 ± 52.06	83.85 ± 24.56	0.221

*Note:* Data are presented as mean ± standard deviation for continuous variables and *n* (%) for categorical variables. Between‐group differences were assessed using independent‐samples *t*‐tests for continuous variables and Pearson chi‐squared tests for categorical variables. PSE, post‐stroke epilepsy; SUA, serum uric acid (μmol/L); NIHSS, National Institutes of Health Stroke Scale; Rangelobe, count of involved cortical lobes (0–5). Urea, serum urea (mmol/L); Creatinine, serum creatinine (μmol/L). *p*‐values are two‐sided; statistical significance was set at *p* < 0.05.

A total of 21,459 patients with acute ischemic stroke were included in the analysis, and 936 (4.36%) developed post‐stroke epilepsy (PSE) within one year. The cohort had a mean age of approximately 66 years and was balanced by sex (~50% male). Compared with patients without PSE, those with PSE had higher baseline NIHSS scores and were more likely to have cortical involvement (both *p* < 0.001). Admission SUA levels did not differ materially in crude comparisons (*p* = 0.632), supporting the use of nonlinear modeling and interaction testing rather than simple linear contrasts.

**FIGURE 2:**
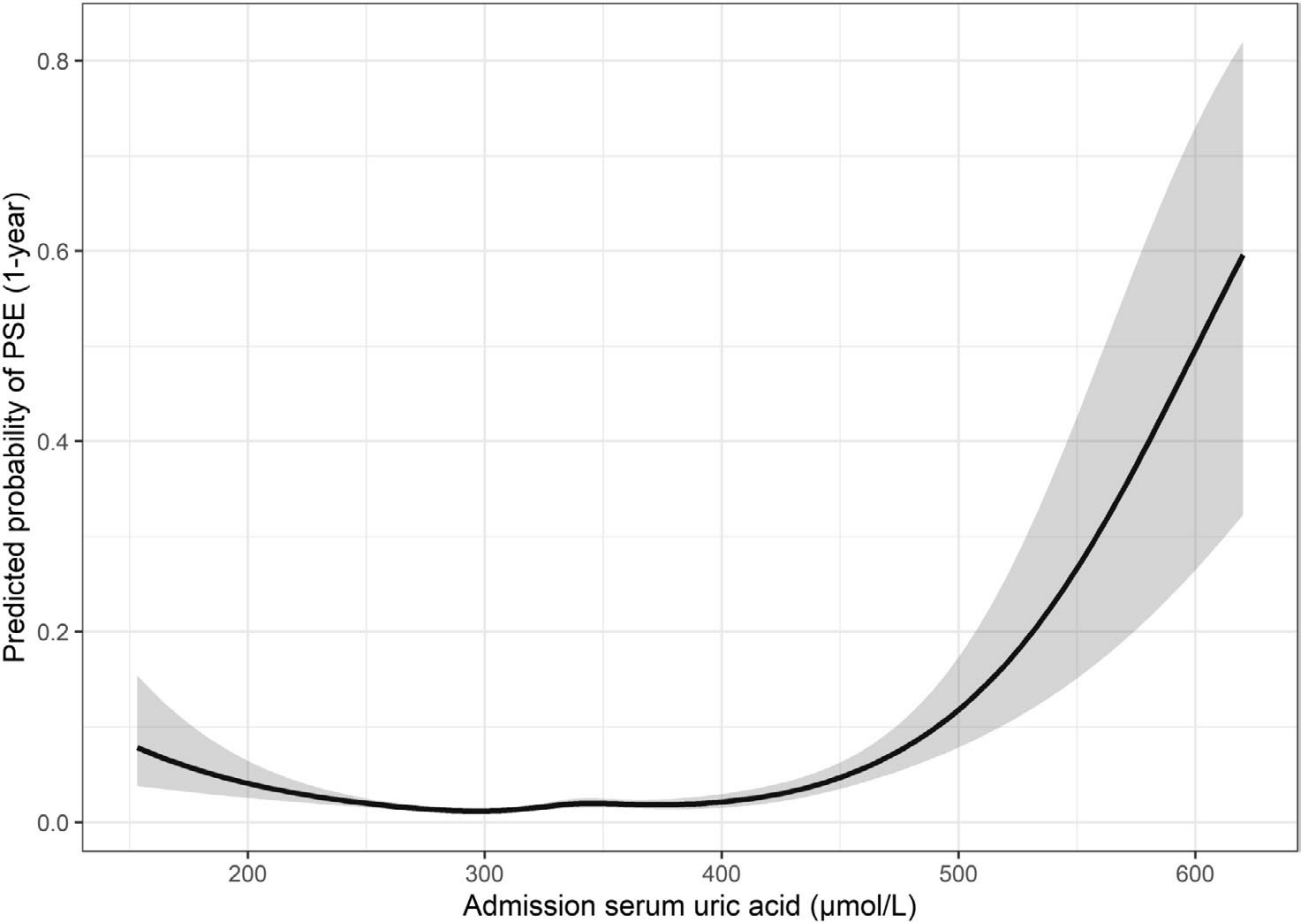
Nonlinear association between admission serum uric acid and 1‐year post‐stroke epilepsy risk. Restricted cubic spline curve showing the adjusted predicted probability of 1‐year post‐stroke epilepsy (PSE) as a function of admission serum uric acid (SUA) in the overall cohort. The solid line represents the model‐estimated association, and the shaded area indicates the 95% confidence interval. Predicted probabilities were estimated from the fully adjusted model at fixed covariate values (continuous covariates at their medians, categorical covariates at reference levels) and should not be interpreted as marginal cohort incidence rates. Estimates at extreme SUA values may be unstable due to sparse data.

Nonlinear Association Between SUA and Post‐Stroke Epilepsy (PSE) (Figure 2). Restricted cubic spline analysis demonstrated a statistically significant nonlinear association between SUA and Post‐Stroke Epilepsy (PSE) (P for nonlinearity < 0.001). The adjusted spline curve suggested a non‐monotonic relationship between SUA and the predicted probability of PSE. Specifically, the risk of PSE increased beyond approximately 450 μmol/L, while lower‐to‐moderate SUA levels were associated with relatively stable or modestly varying risk. When SUA was modeled as a simple linear term, the association appeared attenuated compared with the spline model, indicating that linear modeling would underestimate the complexity of the relationship. The nonlinear pattern persisted after adjustment for the prespecified covariate set (age, sex, NIHSS score, cortical involvement, urea, and creatinine).

### 3.2 Multivariable Logistic Regression

Analysis (Table 2) In the fully adjusted spline model, admission SUA demonstrated a strong overall association with one‐year PSE risk with clear evidence of nonlinearity (joint Wald χ^2^ test *p* < 0.001; nonlinearity component *p* < 0.001). The continuous sex × SUA interaction term from the fully adjusted multivariable logistic regression model had β = −0.00786 (log‐OR per 1 μmol/L increase in SUA; SE = 0.00134; *p* = 3.99 × 10^−9^), corresponding to an OR per 1 μmol/L shift = 0.992 (95% CI 0.990–0.995), or equivalently an OR per clinically meaningful 50 μmol/L shift = 0.675 (95% CI 0.592–0.769). This continuous spline‐based interaction, which is unaffected by tertile cut‐point choice, is the primary and most reliable evidence for sex heterogeneity in the SUA–PSE relationship. These results indicate that the exposure–response pattern differs by sex and cannot be adequately summarized by a single linear effect estimate.

**TABLE 2:** Wald χ^2^ tests for spline terms and sex interaction.

Effect	Chi‐square	df	*p*
SUA (Overall)	291.79	6	< 0.001
Nonlinearity (SUA)	260.98	4	< 0.001
Sex × SUA Interaction	89.04	3	< 0.001
Nonlinear interaction component (Sex × SUA nonlinear terms)	18.45	2	0.0002

*Note:* Wald chi‐squared tests were derived from the fully adjusted multivariable logistic regression model incorporating restricted cubic splines for serum uric acid (SUA) with four knots placed at the 5th, 35th, 65th, and 95th percentiles, fitted using the rms package (R version 4.5.2). The overall SUA effect (df = 6) decomposes into a linear component and a nonlinearity component (df = 4). The sex × SUA interaction term was added as a formal interaction test; the nonlinear interaction component isolates the contribution of the nonlinear spline terms to the interaction. df, degrees of freedom.

### 3.3 Sex Interaction and Stratified Analyses (Figure 3, Table 3)

**FIGURE 3:**
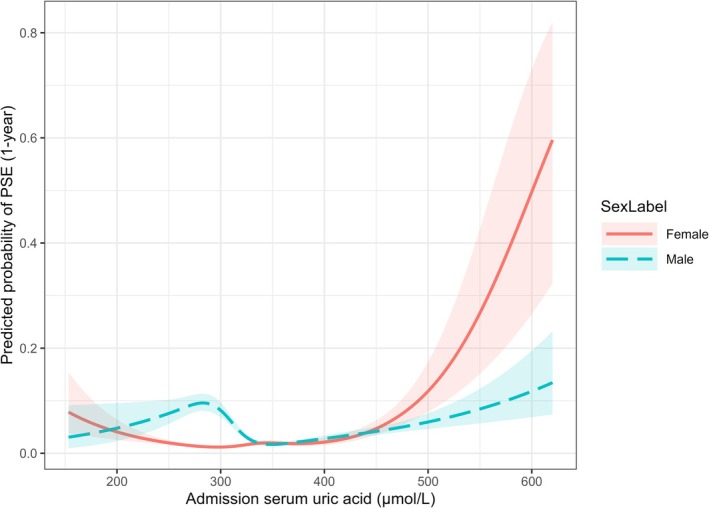
Sex‐stratified associations between admission serum uric acid and 1‐year post‐stroke epilepsy. Sex‐stratified restricted cubic spline curves showing the adjusted predicted probability of 1‐year post‐stroke epilepsy (PSE) as a function of admission serum uric acid (SUA) separately in women and men. Shaded areas indicate 95% confidence intervals. Predicted probabilities were estimated at fixed covariate values and should not be interpreted as marginal incidence rates. The divergent curve shapes illustrate the statistically significant sex‐by‐SUA interaction (*P* = 0.0002).

**TABLE 3:** Sex‐stratified association of SUA tertiles with one‐year PSE.

Sex	SUA Tertile	N (Events)	Incidence (%)	Adjusted OR (95% CI)	*p*
Female	Low	5,458 (190)	3.48%	1.00 (Reference)	—
Female	Mid	3,218 (86)	2.67%	1.039 (0.790–1.366)	0.786
Female	High	1,940 (88)	4.54%	2.331 (1.705–3.186)	< 0.001
Male	Low	1,719 (217)	12.62%	1.00 (Reference)	—
Male	Mid	3,911 (143)	3.66%	0.298 (0.233–0.381)	< 0.001
Male	High	5,213 (212)	4.07%	0.361 (0.283–0.462)	< 0.001

*Note:* Adjusted odds ratios (ORs) and 95% confidence intervals (CIs) were estimated from a multivariable logistic regression model adjusted for age, NIHSS score, cortical involvement (binary indicator and involved‐lobe count), urea, and creatinine. SUA tertile boundaries were defined based on the overall cohort distribution and applied uniformly across both sexes: Low < 316.4, Mid 316.4–356.1, High ≥ 356.1 μmol/L. Because overall tertile cut‐points produce imbalanced sex‐specific strata, a sensitivity analysis using sex‐specific tertile boundaries was performed (Table S9). The Low tertile within each sex served as the reference category (OR = 1.00). Incidence (%) denotes the observed proportion of PSE events within each sex‐tertile stratum. PSE, post‐stroke epilepsy; SUA, serum uric acid; OR, odds ratio; CI, confidence interval; NIHSS, National Institutes of Health Stroke Scale.

A statistically significant interaction between SUA and sex was observed (overall *p* < 0.001; nonlinear component *p* = 0.0002). In sex‐stratified spline analyses, women exhibited a more pronounced J‐shaped pattern, with a steeper increase in predicted PSE risk at higher SUA concentrations, whereas men showed a comparatively flatter risk curve with more modest variation across most of the SUA range. Divergence between the curves was most evident toward the upper tail of SUA, where uncertainty also increased due to fewer observations.

### 3.4 Sensitivity Analysis: Sex‐Specific Tertile Cut‐Points (Table S9)

The sex‐specific tertile sensitivity analysis (Table S9) confirmed the elevated PSE risk in women with high SUA (sex‐specific High vs. Low OR = 1.50, 95% CI 1.13–1.98, *p* = 0.005), albeit attenuated relative to the overall‐tertile estimate (OR = 2.33). In men, the apparent protective association at high SUA using overall tertiles (OR = 0.36, *p* < 0.001) was substantially attenuated and no longer statistically significant with sex‐specific cut‐points (OR = 0.84, 95% CI 0.65–1.07, *p* = 0.16), indicating that this finding is a stratification‐method artifact driven by an extremely low‐SUA male subgroup rather than a genuine protective biological effect. The overall‐tertile analysis is reported for comparability with prior literature; the sex‐specific tertile analysis and the continuous spline interaction are the primary and most robust tests of the sex‐dependent association.

### 3.5 Sensitivity Analysis: NIHSS ≤ 15 Restriction (Table S5)

To assess whether the observational findings were driven by patients with severe stroke (NIHSS > 15), we repeated the primary analyses restricted to patients with admission NIHSS ≤ 15. The nonlinear SUA–PSE association was preserved (P for nonlinearity < 0.001), and the nonlinear sex × SUA interaction component remained highly significant (*p* = 0.0002), confirming that the sex‐dependent pattern is not attributable to severe‐stroke cases. Sex‐stratified tertile estimates were directionally consistent with the primary analysis (female High vs. Low OR = 3.77, 95% CI 2.68–5.31, *p* = 2.74 × 10^−14^; male High vs. Low OR = 0.43, 95% CI 0.33–0.55, *p* = 1.09 × 10^−10^; Table S5).

### 3.6 Supportive Model Discrimination (Figure 4)

**FIGURE 4:**
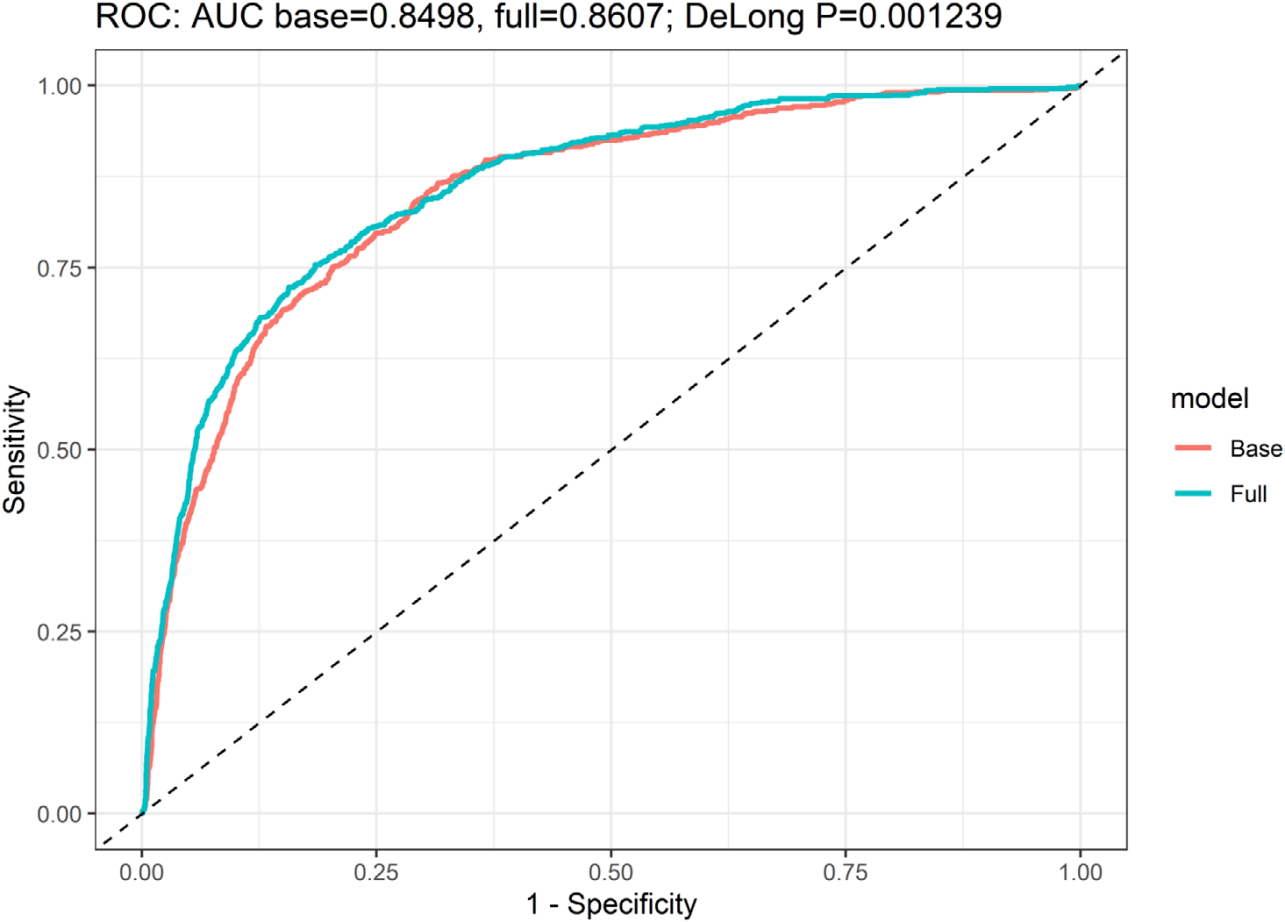
ROC curves comparing the base clinical model and serum uric acid‐augmented model (supportive). Receiver operating characteristic (ROC) curves comparing the discrimination of the base clinical model and the model additionally including serum uric acid (SUA), presented as a supportive analysis. The area under the curve (AUC) was 0.8498 for the base model and 0.8607 for the SUA‐augmented model (DeLong test *p* = 0.001239).

The base clinical model demonstrated good discrimination, and the addition of SUA produced only a modest increase in AUC. Bootstrap‐corrected discrimination remained stable, suggesting minimal overfitting. These results are presented as supportive analyses rather than the primary basis for inference.

### 3.7 Supportive Calibration (Figure 5)

**FIGURE 5:**
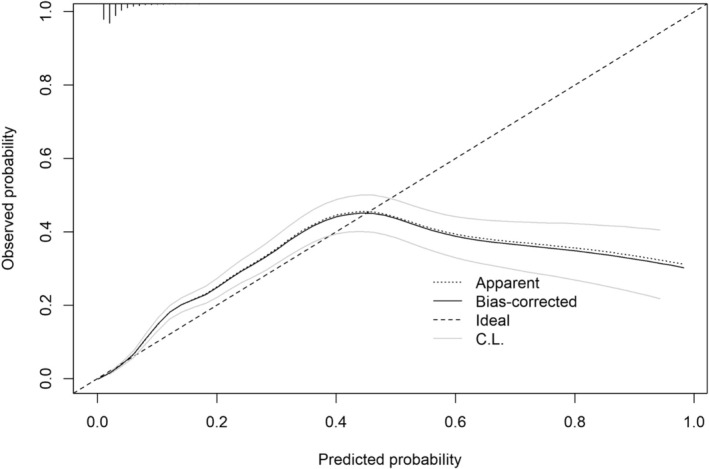
Calibration plots for the base clinical model and the SUA‐augmented model (supportive analysis). Calibration plots for the base clinical model and the model additionally including serum uric acid (SUA), showing observed vs. predicted probability of 1‐year post‐stroke epilepsy (PSE). Apparent, bias‐corrected, and ideal calibration curves are shown, with confidence limits. Note: The extended probability range on the x‐axis reflects the model‐predicted scale at fixed covariate values; the observed event rate in the full cohort is 4.36%. Calibration assessment should be interpreted in the context of supportive rather than primary analyses.

Calibration plots demonstrated good agreement between predicted and observed probability for both the base and SUA‐augmented models. Although calibration intercept and slope may reach statistical significance in a large sample, visual calibration assessment remained acceptable and was interpreted as supportive evidence.

### 3.8 Supportive Decision Curve Analysis (Figure 6)

**FIGURE 6:**
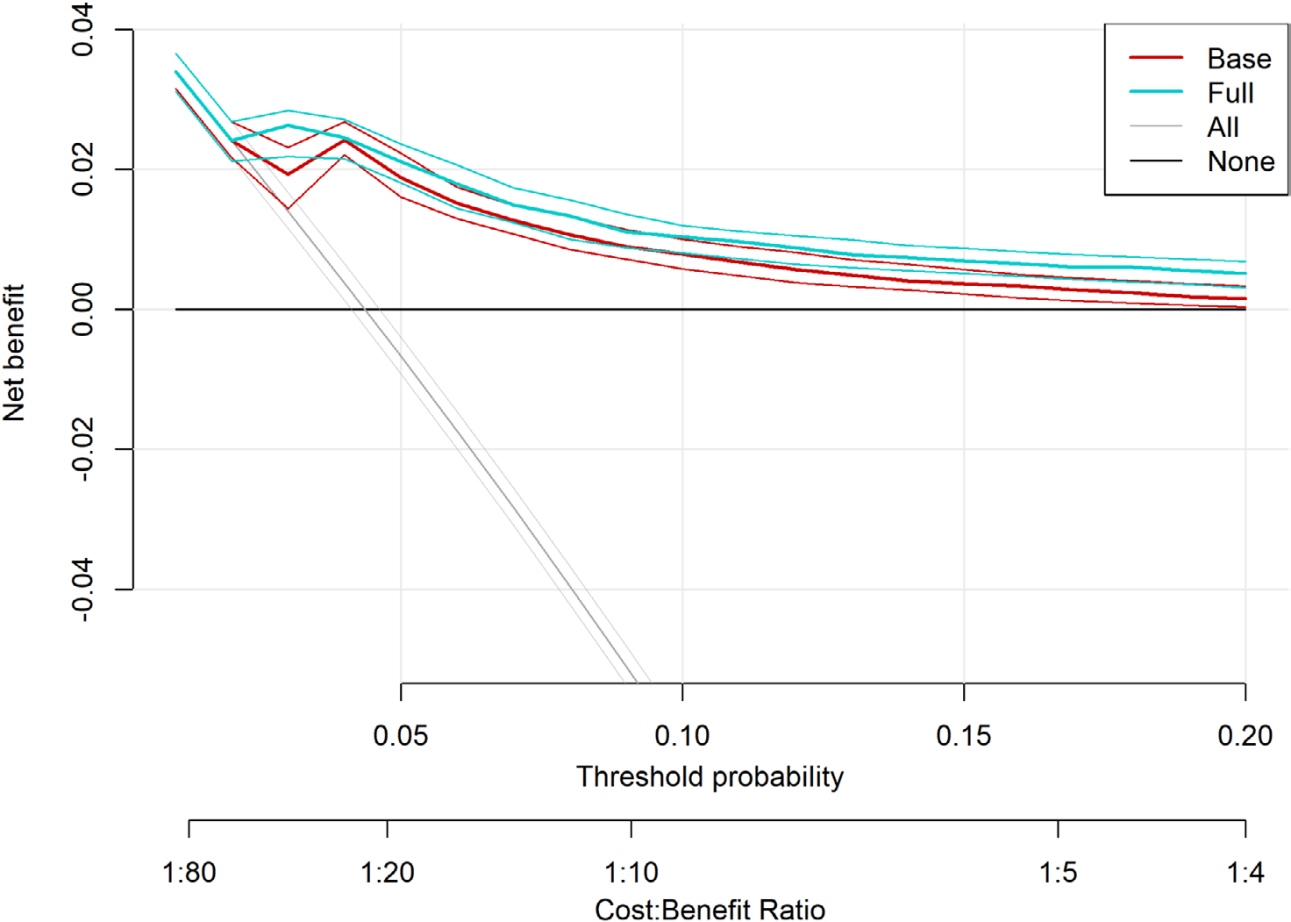
Decision curve analysis: Base clinical model vs. serum uric acid‐augmented model (supportive). Decision curve analysis (DCA) comparing net benefit across threshold probabilities for the base clinical model and the model additionally including serum uric acid (SUA), presented as a supportive analysis. ‘Treat all’ and ‘treat none’ strategies are shown as reference lines.

Decision curve analysis suggested a modest incremental net benefit for the SUA‐augmented model across clinically relevant threshold ranges. Given the small change in global discrimination, these findings were interpreted primarily as supportive evidence rather than a major gain in predictive performance.

### 3.9 Mendelian Randomization

Two‐sample MR analysis identified 323 genetic instruments for SUA after LD pruning; 299 remained after harmonization with the epilepsy outcome GWAS. The IVW method found no evidence of a shared lifelong genetic causal architecture between serum urate and epilepsy susceptibility (OR = 1.043, 95% CI 0.926–1.174, *p* = 0.487; Table S6). Results were consistent across all sensitivity analyses: MR‐Egger (OR = 1.057, 95% CI 0.867–1.289, *p* = 0.582), weighted median (OR = 1.056, 95% CI 0.866–1.289, *p* = 0.589), and weighted mode (OR = 1.003, 95% CI 0.296–3.393, *p* = 0.996). The MR‐Egger intercept test showed no significant horizontal pleiotropy (intercept = −0.0004, SE = 0.00225, *p* = 0.865). Cochran's Q test indicated no significant heterogeneity across instruments for either IVW (Q = 317.80, df = 298, *p* = 0.206) or MR‐Egger (Q = 317.77, df = 297, *p* = 0.195; Table S7). Leave‐one‐out analysis confirmed that no single instrument disproportionately drove the overall estimate, and the funnel plot appeared approximately symmetric. Per‐SNP scatter (Figure S1), forest (Figure S2), and funnel plots (Figure S3) are presented in the Supporting Information. These findings do not support a shared lifelong genetic causal architecture between serum urate and general epilepsy susceptibility, and do not exclude acute post‐stroke, context‐specific mechanisms operating outside the scope of MR. Complete MR results, including scatter, forest, funnel, and leave‐one‐out plots, are presented in the Supporting Information (Tables S6–S7, Figures S1–, S4). The wider confidence interval of the weighted mode estimate (OR = 1.003; 95% CI 0.296–3.393) reflects the lower statistical efficiency of mode‐based estimators under the null, as such estimators concentrate information on the modal cluster of instrument‐specific Wald ratios, rather than instability of the causal estimate itself.

## 4 Discussion

### 4.1 Principal Findings

Our study provides three principal findings. First, admission SUA shows a robust non‐linear observational association with 1‐year PSE in women, confirmed by both spline and sex‐specific tertile analyses (OR = 1.50, 95% CI 1.13–1.98, *p* = 0.005). Second, the apparent protective association at high SUA in men (OR = 0.36 with overall tertiles) is not a robust biological finding but a stratification artifact driven by an extremely low‐SUA male subgroup; the sex‐specific tertile analysis yielded no significant male‐specific protection (OR = 0.84, *p* = 0.16). Third, Mendelian randomization against the largest available cross‐ancestry urate GWAS (*N* = 1,029,323) and ILAE epilepsy GWAS (29,944 cases) yielded a null result (IVW OR = 1.043, *p* = 0.487), indicating no shared lifelong genetic causal architecture between urate and general epilepsy susceptibility. Following the triangulation framework [33], the coherent pattern of (i) positive observational association in women, (ii) null MR, and (iii) significant sex × SUA interaction supports the interpretation of admission SUA as a sex‐dependent metabolic surrogate of PSE risk rather than a direct causal agent.

### 4.2 Comparison With Previous Studies

A recent cohort analysis by Liu et al. [24] using the same publicly available AIS dataset (*n* = 21,459) reported a U‐shaped observational association between admission SUA and post‐stroke epilepsy, with evidence of sex‐stratified variation. Our study extends that work by integrating Mendelian randomization, formal sex‐interaction testing, and a causal‐interpretive framework. The broader observational literature on SUA and stroke outcomes has been inconsistent, with some studies reporting protective associations [6, 7] and others linking elevated SUA to increased risk [9, 34]. Meta‐analyses have yielded conflicting conclusions [12, 13, 35]. Several large cohort studies have themselves reported nonlinear (U‐shaped, J‐shaped, or threshold‐shaped) associations between admission SUA and post‐stroke mortality or functional outcomes [36, 37, 38], indicating that the SUA–stroke literature has progressively recognized flexible nonlinear modeling as essential for capturing the underlying risk gradient. Neither Liu et al. nor prior studies formally addressed causality using genetic instruments or performed rigorous sex‐interaction testing. Our MR analysis is among the first to integrate genetic causal inference with observational interaction analysis on this question, substantially extending the interpretive framework beyond what was achievable in observational designs alone.

The null MR result challenges simple causal interpretations of the U‐shaped observational association. Three mechanisms may explain why observational associations emerge without genetic causality. First, SUA may be a downstream marker of metabolic syndrome severity rather than a direct epileptogenic cause [14, 15, 39]. Second, acute admission SUA captures transient pathophysiological states (dehydration, renal impairment, inflammatory response) not reflected by lifelong genetic exposure. Third, sex‐specific hormonal confounding—particularly estrogen‐mediated regulation of both SUA levels and vascular risk—may generate observational associations that are undetectable in sex‐combined genetic analyses.

### 4.3 Biological Plausibility of the Observational Patterns

Serum urate is a potent endogenous antioxidant at physiological concentrations (∼200–400 μmol/L), where it scavenges peroxyl radicals and peroxynitrite and buffers oxidative stress during ischaemic injury. At supraphysiological concentrations (>∼450 μmol/L), this protective role is progressively overtaken by pro‐oxidant and pro‐inflammatory effects: Urate crystals and soluble urate activate NLRP3 inflammasome signaling, promote endothelial dysfunction through NADPH oxidase–mediated reactive oxygen species generation, impair nitric oxide bioavailability, and amplify blood–brain barrier disruption—all pathways plausibly relevant to the ischaemic brain. These mechanisms together provide a biologically plausible basis for the nonlinear threshold observed near 450 μmol/L, and help explain the inconsistent findings across prior U‐shaped or J‐shaped SUA–stroke outcome studies. We note that this threshold is identified visually from the spline curve rather than estimated as a single parametric inflection point; the primary pre‐specified 4‐knot restricted cubic spline (Harrell's 5th/35th/65th/95th percentiles) is retained as the reference specification.

The striking divergence between women (elevated PSE risk at high SUA) and men (reduced risk) can be understood through sex‐specific metabolic pathways. In women, postmenopausal loss of estrogen‐mediated uricosuric effects leads to SUA elevation that co‐occurs with metabolic syndrome, insulin resistance, and systemic vascular dysfunction [17, 18]—each independently associated with seizure susceptibility. High SUA in women thus serves as a composite marker of metabolic vulnerability rather than a direct epileptogenic agent. In men, higher SUA at admission may reflect preserved antioxidant capacity or better nutritional status, confounding the association with apparent protection. This sex‐specific confounding framework is consistent with the descriptive metabolic profiling data (Table S8, a cross‐sectional comparison that is hypothesis‐supportive rather than mechanistically confirmatory) and the null MR result, supporting the interpretation that SUA's prognostic value derives from its role as a surrogate for sex‐dependent metabolic risk rather than from a direct causal effect on epileptogenesis.

The inconsistency in prior observational studies likely reflects several methodological limitations: Linear‐only modeling that obscures threshold effects; inadequate sex interaction testing; failure to distinguish causal from confounded associations; and smaller sample sizes that limit statistical power for subgroup analyses. The present complementary MR‐plus‐cohort design was specifically constructed to address these limitations.

Our work complements recent efforts to develop PSE risk‐stratification tools using complementary information sources, including clinical scoring algorithms, electroencephalographic biomarkers, neuroimaging features (such as infarct location, cortical involvement, and hemorrhagic transformation), and machine‐learning models built on multidimensional laboratory panels (e.g., the parent‐cohort machine‐learning model of Liu J et al. [26]). Unlike these prediction‐focused frameworks, our analysis pursues a biomarker‐interpretation rather than a prediction‐competition aim: We ask whether admission SUA, as a readily available laboratory variable, captures sex‐dependent metabolic risk information that is complementary to the imaging and electrophysiological substrates covered by these prediction tools.

Our null MR result contrasts with the positive signal reported by Wang & Chen [40]. A structured methodological comparison clarifies the divergence: Their analysis used 31 SNPs from an older European‐ancestry urate GWAS and an older 15,212‐case epilepsy GWAS without F‐statistic reporting, while our analysis used 299 SNPs from the Cho et al. 2024 cross‐ancestry urate GWAS (*N* = 1,029,323) and the ILAE 2023 epilepsy GWAS (29,944 cases) with mean F = 169.5 and 0/299 weak instruments. The substantially greater instrument strength, broader population coverage, larger outcome sample, and formal instrument‐strength diagnostics in our analysis support interpreting our null as the more robust current estimate under the improved data resources, rather than as a refutation of the earlier analysis. Definitive resolution will require sex‐stratified urate GWAS and PSE‐specific outcome GWAS at a consortium scale.

### 4.4 Clinical Implications

Our findings carry hypothesis‐generating rather than directly actionable clinical implications. First, the null MR estimate does not support treatment inference based on a shared genetic causal architecture alone; this is consistent with the absence of benefit signals in the URICO‐ICTUS trial of acute intravenous uric acid supplementation [41], although the URICO‐ICTUS test of acute neuroprotective supplementation is mechanistically distinct from the MR test of lifelong genetically proxied exposure, and their directional concordance is informative but not a formal replication. Second, the significant sex × SUA interaction (*p* = 3.99 × 10^−9^) means that a single sex‐averaged SUA threshold is not appropriate for clinical decision‐making. In women, elevated admission SUA (the highest sex‐specific tertile) is a candidate marker for closer PSE surveillance, pending external validation in independent prospective cohorts. In men, the apparent protective association at high SUA using overall tertiles is not a robust biological finding and should not guide clinical decisions. Third, although the MR analysis does not support a shared lifelong genetic causal architecture between serum urate and general epilepsy susceptibility, it provides modest but consistent incremental predictive net benefit on decision curve analysis in the 3%–8% predicted‐risk band most clinically relevant for PSE; outside this range, the added value of SUA is negligible. As a downstream metabolic surrogate, admission SUA may enrich existing sex‐stratified risk stratification models as a readily available, low‐cost laboratory variable, though external validation is required before any clinical recommendation. The increment in discrimination (ΔAUC = 0.011) is statistically significant but small, and routine SUA measurement as a standalone PSE screening tool is not warranted by the present evidence.

### 4.5 Strengths and Limitations

This study has several strengths: The large sample (*n* = 21,459) provided adequate power for nonlinear and interaction analyses; RCS modeling captured threshold effects missed by linear approaches; formal Wald interaction testing provided rigorous evidence for sex‐dependent heterogeneity; and the complementary MR design addressed causality beyond observational inference. Several limitations warrant explicit discussion. First, the Mendelian randomization used general epilepsy susceptibility as the outcome GWAS because no PSE‐specific GWAS is currently available at a consortium scale; the MR inference is therefore indirect and limited to the lifelong genetic dimension of the SUA–epilepsy relationship. The null MR does not exclude acute post‐stroke, context‐specific epileptogenic mechanisms, including blood–brain barrier disruption, acute reperfusion‐associated oxidative stress, and peri‐infarct inflammation, that operate on biological timescales outside the scope of lifelong genetic instruments. Second, the exposure GWAS is cross‐ancestry, but the outcome GWAS is predominantly European; our observational cohort is Chinese. Cross‐ancestry transportability of the genetic instruments and the specific SUA thresholds may not extend directly to other populations. Third, the publicly available de‐identified source dataset did not contain variables capturing Trial of Org 10,172 in Acute Stroke Treatment (TOAST) stroke etiology, intravenous thrombolysis or endovascular thrombectomy (reperfusion therapy), urate‐lowering therapy (allopurinol, febuxostat), thiazide diuretics, angiotensin‐II receptor blockers, admission fluid resuscitation or hydration status, dietary purine intake, post‐stroke infection, serial hepatic and renal function measurements, family history of epilepsy, stroke recurrence during follow‐up, or post‐admission antiseizure medication (ASM) exposure. Each of these factors is plausibly related to both SUA and PSE risk, and their absence represents a residual confounding limitation; we were unable to perform sensitivity analyses excluding patients receiving reperfusion or urate‐lowering treatment because these fields are not recorded in the source dataset. Fourth, the outcome was ascertained as a 1‐year binary indicator; time‐to‐event analyses within the 1‐year window were not possible. Fifth, predictive model validation was internal only (1000 bootstrap replicates); external validation in independent, ancestry‐diverse, prospective cohorts with PSE phenotyping by a consistent definition is an essential prerequisite for any clinical application. Sixth, sex‐stratified two‐sample MR was not feasible because sex‐stratified urate GWAS summary statistics of comparable sample size are not currently publicly available; formal genetic sex‐dependence therefore remains an open question pending release of such resources. Seventh, the cohort is multicenter but hospital‐based and Chinese; dietary purine intake, alcohol consumption patterns, urate‐lowering therapy prevalence, and the distribution of metabolic comorbidities (diabetes, hyperuricemia) differ markedly across populations, limiting direct generalizability to European, South Asian, or African populations. For the most clinically relevant unmeasured exposures, the plausible direction of residual confounding is as follows: Urate‐lowering therapy (allopurinol, febuxostat) would be expected to lower measured SUA in metabolically high‐risk individuals and therefore bias the observed female SUA–PSE association toward the null, implying that our female high‐SUA effect estimate may be conservative; thiazide diuretics raise SUA and may independently influence electrolyte‐mediated seizure risk, so their absence may introduce bidirectional confounding; admission hydration status would primarily contribute non‐differential measurement noise biasing toward the null; and reperfusion therapy may influence both acute SUA trajectories (through reperfusion‐associated oxidative stress) and seizure risk (through tissue salvage and blood–brain barrier dynamics), constituting a structural confounder whose net direction we cannot determine.

## 5 Conclusion

Among patients with acute ischemic stroke, admission serum uric acid in the highest sex‐specific tertile was associated with an adjusted 1.50‐fold increase (95% CI 1.13–1.98, *p* = 0.005) in 1‐year post‐stroke epilepsy risk in women—a robust estimate confirmed by the continuous sex × SUA interaction (β = −0.00786, *p* = 3.99 × 10^−9^). The overall‐tertile estimate (OR = 2.33), obtained when the same threshold was applied uniformly across sexes, is provided for completeness but must be interpreted in the context of the imbalanced strata produced by pooled cut‐points. The apparent protective association at high SUA in men (OR = 0.36 with overall tertiles) is not robust to sex‐specific stratification and likely reflects a vulnerability phenotype in the very‐low‐SUA male subgroup. Mendelian randomization against the largest available urate and epilepsy GWAS yielded a null result, indicating no shared lifelong genetic causal architecture between SUA and general epilepsy susceptibility; this null does not exclude acute post‐stroke, context‐specific mechanisms that operate on biological timescales outside the scope of MR. These findings are hypothesis‐generating: Confirmation in independent, ancestry‐diverse, prospectively ascertained cohorts with PSE phenotyping by a consistent definition is required before any clinical recommendation can be made.

## 6 Reporting Standards

This study was reported in accordance with the Strengthening the Reporting of Observational Studies in Epidemiology (STROBE) guidelines for the cohort component and the STROBE‐MR framework [29] for the Mendelian randomization component.

Completed STROBE and STROBE‐MR checklists are provided as Additional files 6 and 7, respectively.

## Author Contributions

Conceptualization: Yan Cheng, Yunzhou Yang. Methodology: Yan Cheng, Yunzhou Yang. Formal analysis: Yan Cheng. Data curation: Yan Cheng. Writing – original draft: Yan Cheng. Writing – review and editing: Ji Li, Yunzhou Yang. Supervision: Yunzhou Yang. All authors approved the final manuscript.

## Funding

This secondary analysis received no specific external funding. The parent cohort and data generation were funded by the original investigators as described in the source publication [26].

## Ethics Statement

This study was a secondary analysis of a publicly available, de‐identified dataset obtained from the Dryad Digital Repository (https://doi.org/10.5061/dryad.w0vt4b92c). No additional institutional ethics approval or informed consent was required under local policy for analysis of publicly available anonymized data. The study adhered to the principles of the Declaration of Helsinki.

## Consent

The authors have nothing to report.

## Conflicts of Interest

The authors declare no conflicts of interest.

## Supporting information


**Figure S1:** MR scatter plot: Serum urate → epilepsy susceptibility (N SNPs = 299).

**Figure S2:** Forest plot of MR sensitivity analyses.

**Figure S3:** Funnel plot of MR analysis.

**Figure S4:** Leave‐one‐out analysis plot.

**Data S1:** STROBE Checklist. Completed STROBE checklist for cohort studies.

**Data S2:** STROBE‐MR Checklist. Completed STROBE‐MR checklist 29.

**Table S1:** Variable definitions and derivations.
**Table S2:** Locked model specifications and key metrics.
**Table S3:** Reproducibility checklist.
**Table S4:** Model comparison and reporting framework.
**Table S5:** Sensitivity analysis excluding NIHSS > 15.
**Table S6:** Two‐sample Mendelian randomization: Main results.
**Table S7:** Two‐sample Mendelian randomization: Sensitivity analyses.
**Table S8:** Metabolic and renal characteristics by sex and SUA tertile.
**Table S9:** Sex‐specific tertile sensitivity analysis.
**Table S10:** Instrument‐strength statistics (F‐statistics) for the 299 genetic instruments used in two‐sample Mendelian randomization.
**Table S11:** Sensitivity analysis restricting the cohort to participants aged ≥ 18 years (*n* = 21,457).
**Table S12:** Variance inflation factors (VIF) for all covariates in the multivariable base model.

## Data Availability

The dataset(s) supporting the conclusions of this article is(are) available in the Dryad Digital Repository, https://doi.org/10.5061/dryad.w0vt4b92c [25]. The present work is a secondary analysis of the de‐identified dataset; no additional individual‐level data were collected.
