# Supplementary figures and images for: Serum Uric Acid as a Sex‐Dependent Risk Marker of Post‐Stroke Epilepsy After Acute Ischemic Stroke: Complementary Mendelian Randomization and Cohort Analyses
Source: CNS Neurosci Ther. 2026 Jun 8;32(6):e70970. doi: 10.1002/cns.70970 (PMC13245277; doi:10.1002/cns.70970)

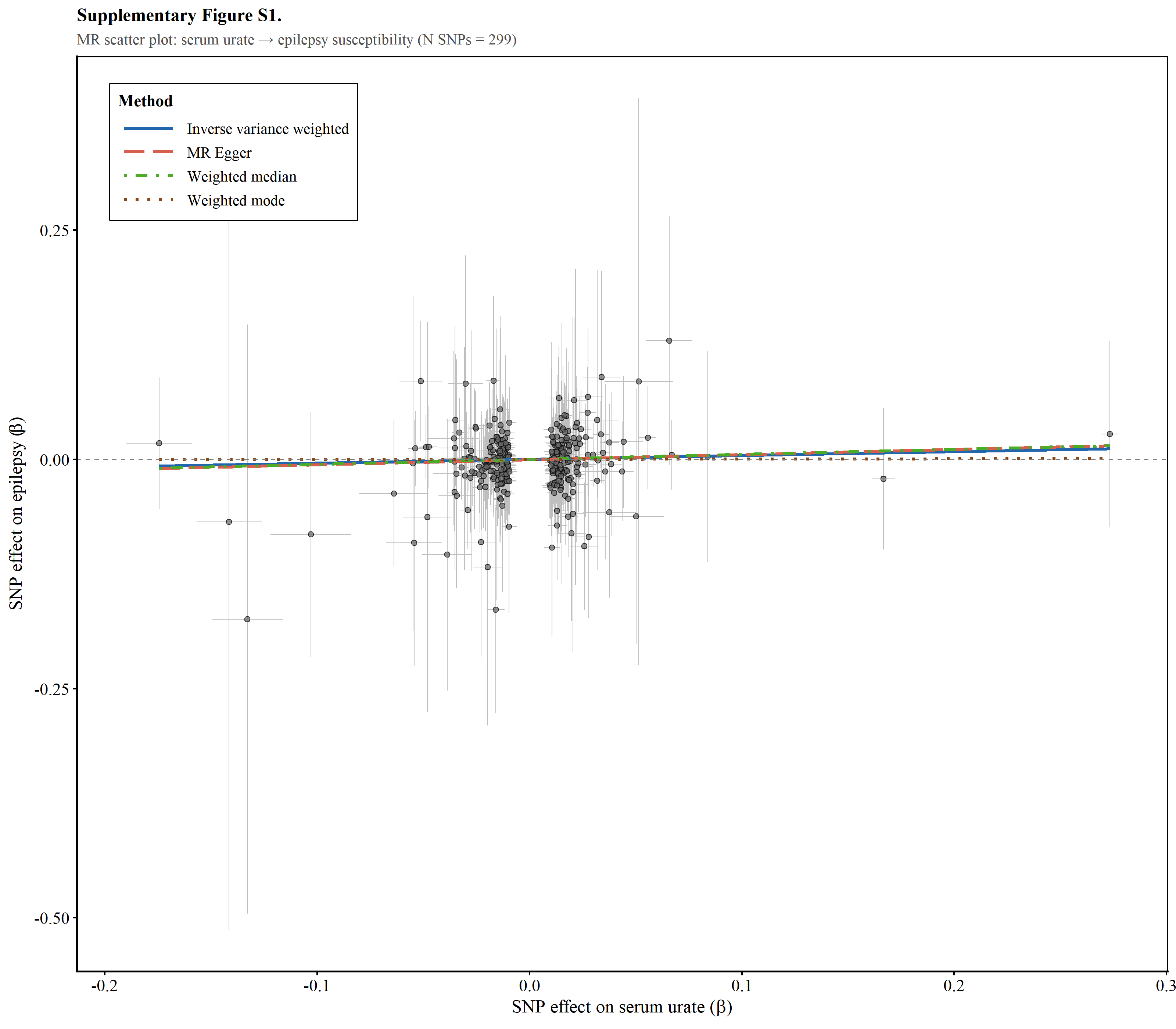

Supplement: Supplementary file 1 — Figure S1: MR scatter plot: Serum urate → epilepsy susceptibility (N SNPs = 299). [file CNS-32-e70970-s004.tif]

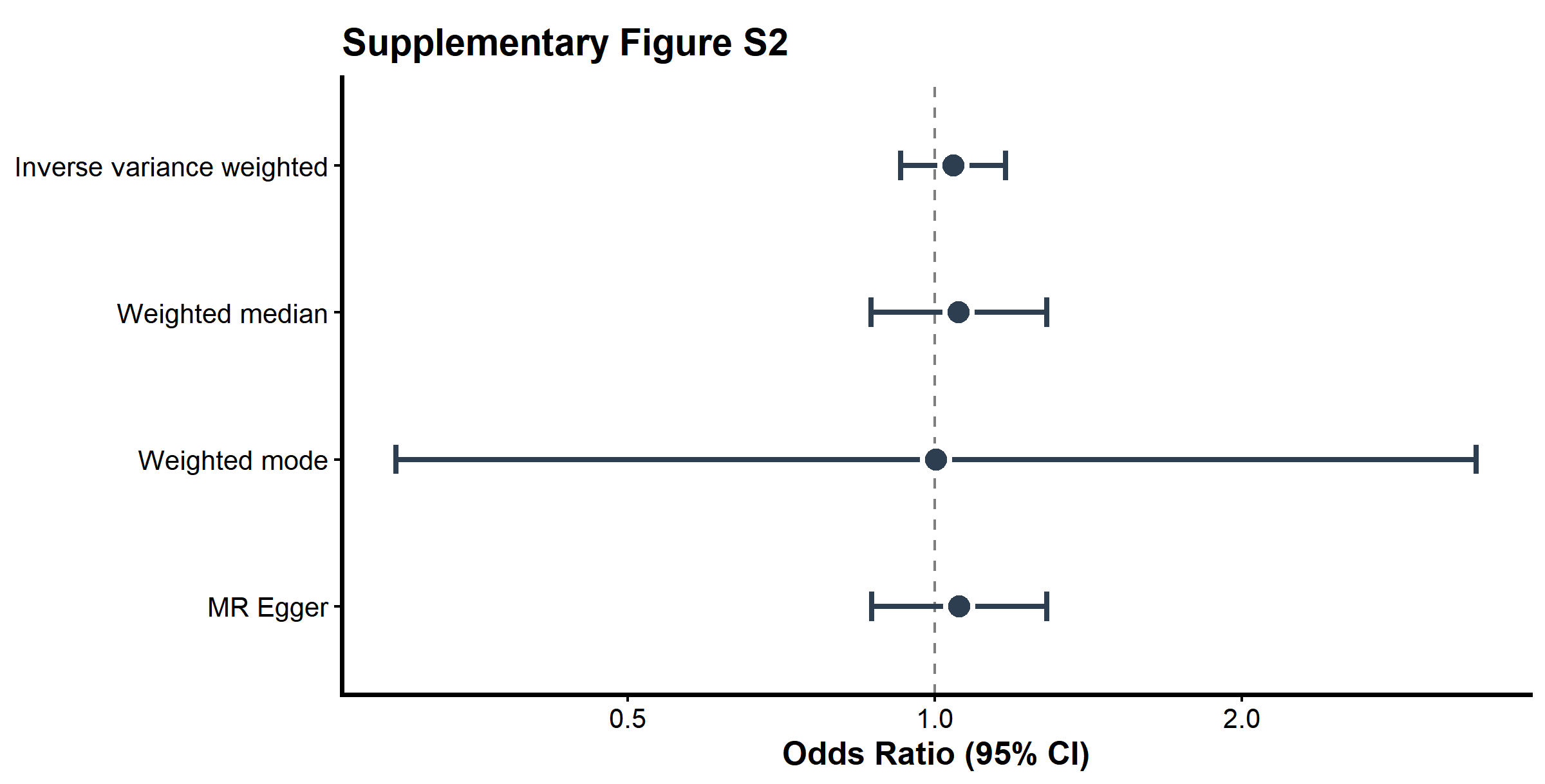

Supplement: Supplementary file 2 — Figure S2: Forest plot of MR sensitivity analyses. [file CNS-32-e70970-s003.tif]

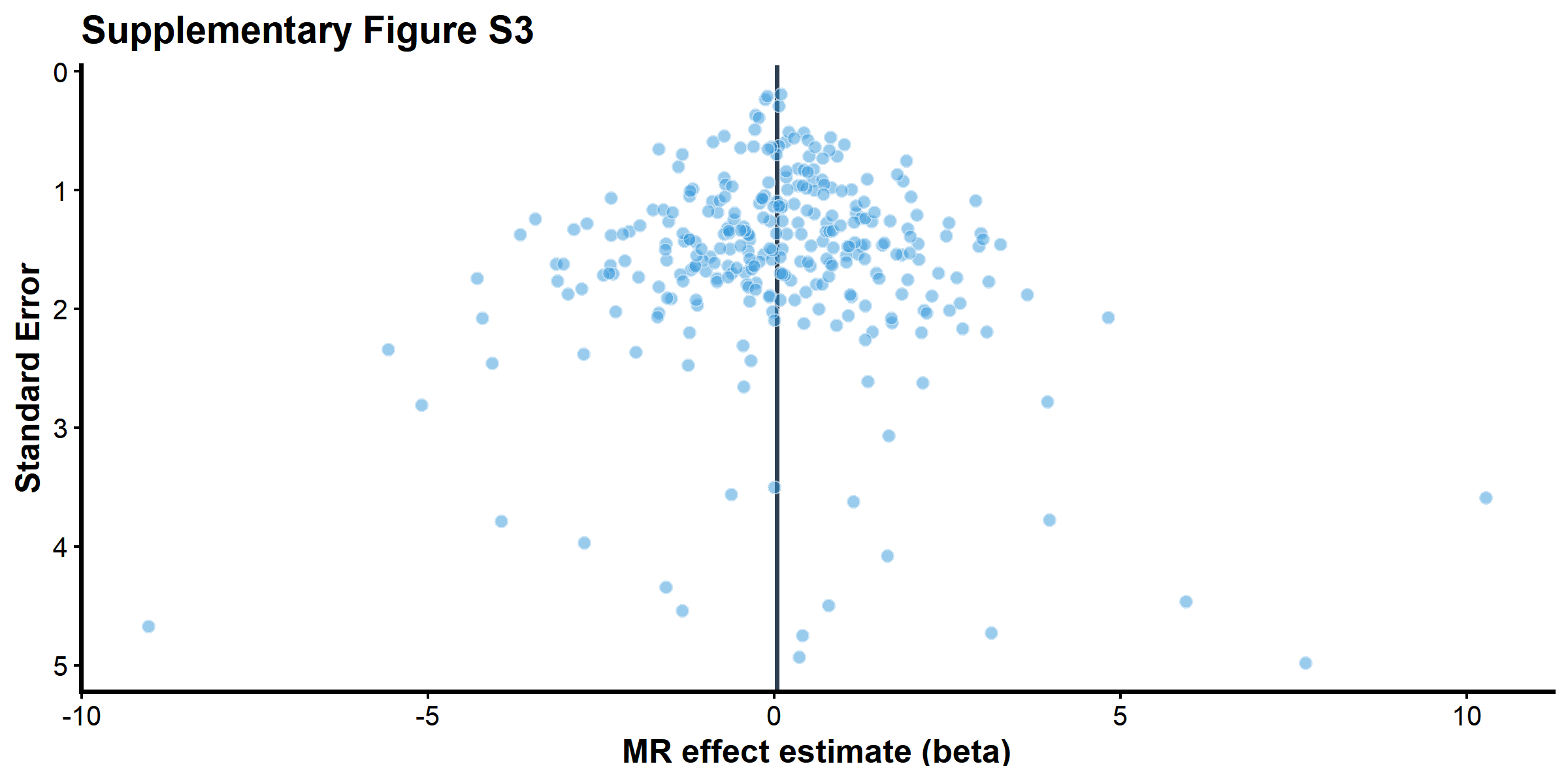

Supplement: Supplementary file 3 — Figure S3: Funnel plot of MR analysis. [file CNS-32-e70970-s001.tif]

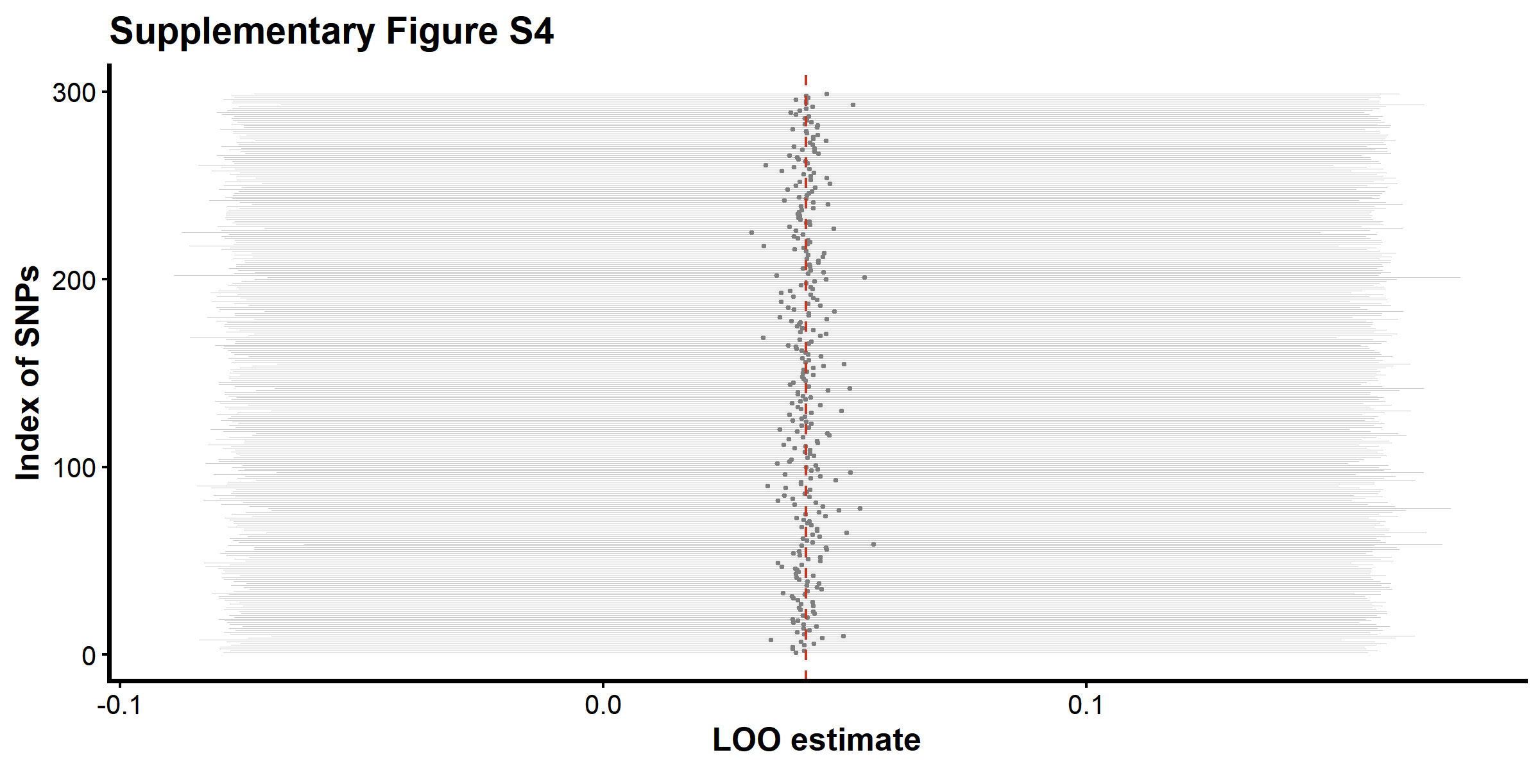

Supplement: Supplementary file 4 — Figure S4: Leave‐one‐out analysis plot. [file CNS-32-e70970-s006.tif]
