# Supplementary material for: Serum Uric Acid as a Sex‐Dependent Risk Marker of Post‐Stroke Epilepsy After Acute Ischemic Stroke: Complementary Mendelian Randomization and Cohort Analyses
Source: CNS Neurosci Ther. 2026 Jun 8;32(6):e70970. doi: 10.1002/cns.70970 (PMC13245277; doi:10.1002/cns.70970)
Supplement: Supplementary file 5 — Data S1: STROBE Checklist. Completed STROBE checklist for cohort studies. [file CNS-32-e70970-s002.docx]

# STROBE-MR Checklist — Manuscript 2001709

**Title:** Serum Uric Acid as a Sex-Dependent Risk Marker of Post-Stroke Epilepsy After Acute Ischemic Stroke: Complementary Mendelian Randomization and Cohort Analyses

**Reference:** Skrivankova VW, Richmond RC, Woolf BAR, et al. *JAMA*. 2021;326(16):1614–1621; *BMJ*. 2021;375:n2233.

**Additional file designation:** Additional file 7

## TITLE AND ABSTRACT

### Item 1. Title and Abstract

**Sub-item 1a.** *Indicate Mendelian randomization (MR) as the study’s design in the title and/or abstract if that is a main focus.*

- **Location in revised manuscript:** Title: “Complementary Mendelian Randomization and Cohort Analyses” explicitly states MR as a design element. Abstract: “Methods” section (revised L. 38–44) identifies the two-sample MR analysis alongside the observational cohort component.
- **Compliance:** ✅

**Sub-item 1b.** *Provide a structured summary including key objectives, methods, data sources, analyses, and conclusions.*

- **Location:** Structured abstract (revised L. 28–55) with Background, Methods, Results, Conclusions sub-headers meeting the journal’s structured abstract requirement.
- **Compliance:** ✅

## INTRODUCTION

### Item 2. Background and rationale

**Sub-item 2a.** *Explain scientific background and rationale for MR approach; state the research question.*

- **Location:** Introduction (revised L. 80–115). Rationale for complementary MR-plus-cohort design and explicit distinction between the observational (admission-SUA-to-1-year-PSE) and MR (lifelong genetic SUA-to-general-epilepsy-susceptibility) questions.
- **Compliance:** ✅

**Sub-item 2b.** *Explain the choice of exposure and outcome, and whether relationships between exposure(s) and outcome(s) have been previously studied.*

- **Location:** Introduction (revised L. 70–95). Explicit positioning against Wang & Chen 2025 and Liu D et al. 2026. Discussion (revised L. 391–404) for detailed comparison.
- **Compliance:** ✅

### Item 3. Objectives

*State specific objectives, including prespecified causal hypotheses.*

- **Location:** Introduction, final paragraph (revised L. 108–130). Two aims explicitly separated: (i) observational sex-dependent admission-SUA to 1-year PSE association; (ii) lifelong genetic MR between urate and general epilepsy susceptibility. Prespecified hypothesis: **null MR + positive observational + significant sex interaction = sex-dependent metabolic surrogate rather than causal agent** (triangulation framework).
- **Compliance:** ✅

## METHODS

### Item 4. Study design and data sources

*Present key elements of the study design early; describe cohort and data sources for all MR datasets.*

- **Location:** Methods §“Study Design” (revised L. 120–135) and §“Data Sources” (revised L. 210–235).
- **Summary:**
- Observational cohort: Liu J et al., *eLife* 2024 (Dryad DOI: 10.5061/dryad.w0vt4b92c); N = 21,459 Chinese AIS patients; 936 PSE events within 1 year.
- Exposure GWAS: Cho C et al., *Nat Commun* 2024 (IEU ID: ieu-b-5137); serum urate; N = 1,029,323; cross-ancestry.
- Outcome GWAS: ILAE Consortium on Complex Epilepsies, *Nat Genet* 2023 (IEU ID: ebi-a-GCST90018840); epilepsy; 29,944 cases / 52,538 controls; predominantly European ancestry.
- **Compliance:** ✅

### Item 5. Setting

*Describe the setting, locations, and relevant dates.*

- **Location:** Methods (revised L. 135–145). Observational: multicenter Chinese AIS cohort; patient enrolment 2017–2022. MR: publicly available GWAS summary statistics, dataset versions as of analysis date (2026).
- **Compliance:** ✅

### Item 6. Participants

**Sub-item 6a.** *Cohort: eligibility criteria, sources, methods of selection.*

- **Location:** Methods §“Participants” (revised L. 135–145). Inclusion: adult (≥18 years per parent cohort; see sensitivity analysis L. 136–141), AIS confirmed by imaging, admission SUA available, 1-year PSE outcome ascertained. Exclusion: prior antiseizure medication use; no additional exclusions for the present secondary analysis.
- **Compliance:** ✅

**Sub-item 6b.** *MR: describe each GWAS in terms of population, sample size, ancestry, and any overlap between exposure and outcome GWAS.*

- **Location:** Methods §“Data Sources” (revised L. 210–235) and new Supplementary Methods S2.
- **Sample overlap:** Exposure GWAS (Cho 2024) is cross-ancestry (predominantly European + East Asian + African); outcome GWAS (ILAE 2023) is predominantly European. Non-zero sample overlap in the European component cannot be quantified at the summary-level but is limited by the very different case composition.
- **Compliance:** ✅

### Item 7. Variables — Assumptions

*Clearly define all exposures, outcomes, genetic variants, and the three core MR assumptions (relevance, independence, exclusion restriction).*

- **Location:** Methods §“MR Analysis” (revised L. 210–245). All three core assumptions stated and empirically assessed.
- **Compliance:** ✅

### Item 8. Data sources and measurement

*Describe data sources and measurement methods for all variables.*

- **Location:** Methods §“Measurements” (revised L. 145–170). Admission SUA: standardized hospital laboratory enzymatic colorimetric method. PSE: parent-study ascertainment by trained neurologists per ILAE 2014 definition, within 1-year follow-up window. Full variable definitions in Supplementary Table S1.
- **Compliance:** ✅

### Item 9. Bias

*Describe any efforts to address potential sources of bias.*

- **Location:** Methods §“Statistical Analysis” (revised L. 175–210) and Limitations (revised L. 451–475).
- **Addressed biases:** (i) weak-instrument bias (F-statistic reporting, mean F = 169.5; new Supplementary Table S10); (ii) horizontal pleiotropy (MR-Egger intercept, Cochran’s Q, leave-one-out); (iii) residual confounding (Limitations list TOAST subtype, reperfusion, ULT, hydration, family history, follow-up ASM).
- **Compliance:** ✅

### Item 10. Study size

*Explain how the study size was arrived at.*

- **Location:** Methods §“Participants” (revised L. 135–145). Observational: full eligible cohort (N = 21,459). MR: all SNPs meeting genome-wide significance in the exposure GWAS after LD-pruning and harmonisation (299 SNPs; see Item 13).
- **Compliance:** ✅

### Item 11. Quantitative variables

*Explain how quantitative variables were handled in the analyses.*

- **Location:** Methods §“Statistical Analysis” (revised L. 175–210). SUA analysed as: (a) continuous (restricted cubic splines, 4 knots at the 5th/35th/65th/95th percentiles; Harrell’s default); (b) overall-cohort tertiles (primary for comparability with prior literature); (c) sex-specific tertiles (prespecified sensitivity analysis; Supplementary Table S9 and locked Supplementary Script E).
- **Compliance:** ✅

### Item 12. Statistical methods

**Sub-item 12a.** *Describe all statistical methods, including MR methods and sensitivity analyses.*

- **Location:** Methods §“Statistical Analysis” (revised L. 175–210) and §“MR Analysis” (revised L. 210–245).
- **Methods:** Observational — multivariable logistic regression; restricted cubic splines (rms R package); formal Wald test for sex × SUA interaction (continuous and nonlinear components); sex-specific tertile sensitivity analysis (Supplementary Script E). MR — primary IVW; sensitivity MR-Egger, weighted median, weighted mode (TwoSampleMR v0.7.0). Pleiotropy: MR-Egger intercept test. Heterogeneity: Cochran’s Q test. Robustness: leave-one-out analysis. Internal validation: 1,000 non-parametric bootstrap replicates, not outcome-stratified (justification: large event count; Methods L. 200–207).
- **Compliance:** ✅

**Sub-item 12b.** *Describe any methods used to explore sources of heterogeneity among the MR estimates.*

- **Location:** Methods §“MR Analysis” (revised L. 230–245). Cochran’s Q for IVW and MR-Egger; leave-one-out analysis (Supplementary Figure S4); I²_GX statistic computed as part of mean F-statistic diagnostics.
- **Compliance:** ✅

**Sub-item 12c.** *Describe how missing data were addressed.*

- **Location:** Methods §“Participants” (revised L. 142–144). Participants with complete data for all analytic covariates were retained; missingness was minimal (< 0.5% across all covariates) and addressed by complete-case analysis. No multiple imputation was performed, consistent with the low missingness rate.
- **Compliance:** ✅

**Sub-item 12d.** *If applicable, describe analytical methods to test the assumption of no horizontal pleiotropy.*

- **Location:** Methods §“MR Sensitivity Analyses” (revised L. 230–245). MR-Egger intercept test (intercept = −0.000381, P = 0.865); Cochran’s Q (no significant heterogeneity); leave-one-out analysis. All reported in Supplementary Tables S6, S7 and Supplementary Figures S1–S4.
- **Compliance:** ✅

### Item 13. Instrumental variables

**Sub-item 13a.** *Report genetic variants used as instrumental variables and overall F-statistic or equivalent metric of instrument strength (new Supplementary Table S10).*

- **Location:** Methods §“MR Analysis” (revised L. 220–230) and new Supplementary Table S10. 299 independent SNPs after LD pruning and outcome-GWAS harmonisation. Mean F-statistic = 169.5; median 49.1; minimum 24.2; maximum 20,210.1. Zero weak instruments (F < 10).
- **Compliance:** ✅

**Sub-item 13b.** *Report the number of instruments used and give descriptive statistics of associations with the exposure.*

- **Location:** Supplementary Table S6 (299 SNPs with β, SE, P) and new Supplementary Table S10 (per-SNP F-statistic distribution).
- **Compliance:** ✅

## RESULTS

### Item 14. Descriptive data

*Report the characteristics of study participants and information on exposures and potential confounders.*

- **Location:** Results §“Participants” (revised L. 250–265) and Table 1. N = 21,459; PSE events 936 (4.36%); 10,616 women / 10,843 men; detailed baseline distribution by sex stratum.
- **Compliance:** ✅

### Item 15. Outcome data

*Report outcome data.*

- **Location:** Table 1 and Results §“SUA–PSE Association” (revised L. 250–310). Event counts per sex-tertile stratum in Table 3.
- **Compliance:** ✅

### Item 16. Main results

**Sub-item 16a.** *Give unadjusted and adjusted estimates, where applicable, and their precision.*

- **Location:** Table 2 (adjusted spline estimates), Table 3 (adjusted tertile ORs), Supplementary Table S6 (MR results).
- **Principal observational findings:** Female high tertile OR = 2.33 (95% CI 1.71–3.19, P < 0.001) with overall tertiles; OR = 1.50 (95% CI 1.13–1.98, P = 0.005) with sex-specific tertiles. Male high tertile OR = 0.36 (P < 0.001) with overall tertiles; OR = 0.84 (P = 0.16, **not significant**) with sex-specific tertiles. Sex × SUA continuous interaction: β = −0.00786, P = 3.99 × 10⁻⁹.
- **Principal MR findings:** IVW OR = 1.043 (95% CI 0.926–1.174, P = 0.487); null across all four methods.
- **Compliance:** ✅

**Sub-item 16b.** *Report other analyses done — e.g., analyses of subgroups and interactions, and sensitivity analyses.*

- **Location:** Supplementary Tables S5–S12 (sensitivity analyses: NIHSS ≤ 15 restriction, age ≥ 18 restriction, sex-specific tertiles, VIF collinearity diagnostic). All critical sensitivity analyses reproduced the primary findings.
- **Compliance:** ✅

## DISCUSSION

### Item 17. Key results

*Summarise key results with reference to study objectives.*

- **Location:** Discussion §“Principal Findings” (revised L. 335–350). Three-part summary: (i) non-linear sex-dependent observational association; (ii) null MR; (iii) interpretive synthesis as sex-dependent metabolic surrogate rather than causal agent.
- **Compliance:** ✅

### Item 18. Limitations

*Discuss limitations of the study, taking into account sources of potential bias or imprecision.*

- **Location:** Discussion §“Strengths and Limitations” (revised L. 451–475).
- **Limitations addressed:** (1) general epilepsy GWAS as proxy for PSE; (2) cross-ancestry transportability; (3) residual confounding (TOAST, reperfusion, ULT, hydration, infection, family history, follow-up ASM, hepatic/renal dynamics); (4) internal-only prediction validation (no external cohort); (5) time-to-event data not captured; (6) sex-stratified MR not currently feasible; (7) single-country design.
- **Compliance:** ✅

### Item 19. Interpretation

*Give a cautious overall interpretation, considering objectives, limitations, multiplicity of analyses, and results from similar studies.*

- **Location:** Discussion §“Principal Findings” and §“Clinical Implications” (revised L. 335–440); interpretation explicitly softened to hypothesis-generating language.
- **Compliance:** ✅

### Item 20. Generalisability

*Discuss the generalisability of the study results.*

- **Location:** Discussion §“Strengths and Limitations” (revised L. 466–475). Limited generalisability across ancestries and healthcare systems is explicitly stated; external validation in ancestry-diverse prospective cohorts is identified as a prerequisite for any clinical implementation.
- **Compliance:** ✅

## OTHER INFORMATION

### Item 21. Funding

*Give the source of funding and the role of the funders for the present study and, if applicable, for the original study on which the present article is based.*

- **Location:** Declarations §“Funding” (revised L. 720–730).
- **Statement:** “This secondary analysis received no specific external funding. The parent cohort and data generation were funded by the original investigators as described in the source publication (Liu J et al., *eLife* 2024). The funders had no role in the design, analysis, or interpretation of the present secondary analysis.”
- **Compliance:** ✅

### Item 22. Data and code availability

*Give detailed information on how to access the dataset(s) and statistical code used to generate results.*

- **Location:** Declarations §“Availability of data and materials” (revised L. 712–720) and Supplementary Methods S1.
- **Statement:** “The observational dataset is publicly available from the Dryad Digital Repository (DOI: 10.5061/dryad.w0vt4b92c). The exposure GWAS (ieu-b-5137) and outcome GWAS (ebi-a-GCST90018840) are publicly available from IEU OpenGWAS (https://gwas.mrcieu.ac.uk). Locked analytical scripts (seed = 20260221) are provided as Supplementary Scripts A–E (and new Script F for F-statistic computation, Script G for age ≥18 sensitivity analysis).”
- **Compliance:** ✅

**Checklist prepared by:** Yunzhou Yang, MD, PhD (corresponding author)

**Date:** [Insertion Date]

**Declaration:** All 20 main items and 30 sub-items of the STROBE-MR statement have been addressed in the revised manuscript or accompanying supplementary materials. No items are deemed not applicable to this study design.
