# Supplementary material for: Serum Uric Acid as a Sex‐Dependent Risk Marker of Post‐Stroke Epilepsy After Acute Ischemic Stroke: Complementary Mendelian Randomization and Cohort Analyses
Source: CNS Neurosci Ther. 2026 Jun 8;32(6):e70970. doi: 10.1002/cns.70970 (PMC13245277; doi:10.1002/cns.70970)
Supplement: Supplementary file 6 — Data S2: STROBE‐MR Checklist. Completed STROBE‐MR checklist 29. [file CNS-32-e70970-s005.docx]

**STROBE Checklist — Cohort Study**

*Serum Uric Acid as a Sex-Dependent Risk Marker of Post-Stroke Epilepsy After Acute Ischemic Stroke*

CNS Neuroscience & Therapeutics | Submission 2026

*Reference: von Elm E, et al. The Strengthening the Reporting of Observational Studies in Epidemiology (STROBE) statement: guidelines for reporting observational studies. PLoS Med. 2007;4(10):e296. doi:10.1371/journal.pmed.0040296*

| **Item** | **STROBE Recommendation (Cohort)** | **Location in Manuscript** | **Status** | **Notes** |
| --- | --- | --- | --- | --- |
| **TITLE AND ABSTRACT** | | | | |
| **1a** | Indicate the study's design with a commonly used term in the title or the abstract. | Methods section: 'prespecified secondary analysis of a multicenter AIS cohort' (Study Design and Data Source); Abstract Methods: 'two-sample Mendelian randomization' and 'multicenter retrospective cohort'. | **✅** | Study design clearly labelled as secondary analysis of a cohort study. |
| **1b** | Provide in the abstract an informative and balanced summary of what was done and what was found. | Abstract (Background, Methods, Results, Conclusions) — structured, 4-section format with key quantitative findings (n=21,459; PSE=4.36%; IVW OR=1.043; P interaction=0.0002). | **✅** |  |
| **INTRODUCTION** | | | | |
| **2** | Explain the scientific background and rationale for the investigation being reported. | Introduction ¶1–6: global AIS burden, SUA biology, inconsistent prior evidence, sex-specific heterogeneity, rationale for MR and spline approaches. | **✅** |  |
| **3** | State specific objectives, including any prespecified hypotheses. | Introduction ¶7 (final): two explicit objectives stated — (1) MR causal evaluation; (2) nonlinear/sex-stratified observational analysis. | **✅** |  |
| **METHODS** | | | | |
| **4** | Present key elements of study design at the start of the Methods. | Methods §'Study Design and Data Source': pre-specified secondary analysis of multicenter retrospective cohort; Dryad DOI provided. | **✅** |  |
| **5** | Describe the setting, locations, and relevant dates, including periods of recruitment, exposure, follow-up, and data collection. | Methods §'Data Source and Cohort Description': multicenter tertiary hospitals; enrollment June 2017–June 2022; 1-year follow-up for PSE. | **✅** |  |
| **6** | Give the eligibility criteria, and the sources and methods of selection of participants. Describe methods of follow-up. | Methods §'Study Population': age 18–90, AIS diagnosis, neuroimaging-confirmed; exclusion criteria listed; flowchart in Figure 1. | **✅** |  |
| **7** | Clearly define all outcomes, exposures, predictors, potential confounders, and effect modifiers. Give diagnostic criteria, if applicable. | Methods §'Exposure Variable' and §'Outcome Definition': SUA measured at admission; PSE definition from source dataset (ICD-coded); covariates listed in Statistical Analysis and Table S1. | **✅** |  |
| **8** | For each variable of interest, give sources of data and details of methods of assessment (measurement). Describe comparability of assessment methods if there is more than one group. | Methods §'Exposure Variable': SUA by routine admission biochemistry; outcome ascertained from registry records per parent study protocol; Supplementary Methods S1 and Table S1. | **✅** |  |
| **9** | Describe any efforts to address potential sources of bias. | Methods §'Mendelian Randomization': MR used to address confounding; sensitivity analyses (MR-Egger, weighted median, weighted mode); NIHSS≤15 sensitivity analysis (Table S5). | **✅** |  |
| **10** | Explain how the study size was arrived at. | Supplementary Methods S3 (newly added): secondary analysis of existing registry; no a priori power calculation; post hoc EPV ≈ 93.6; power >99% to detect OR ≥1.30. | **✅** | Added in SupplementaryMaterial. |
| **11** | Explain how quantitative variables were handled in the analyses. If applicable, describe which groupings were chosen and why. | Methods §'Statistical Analysis': SUA modelled continuously with RCS (4 knots, Harrell's algorithm); tertile groupings used for Table 3 and sensitivity analysis; rationale for 4 knots in Supplementary Methods S1. | **✅** |  |
| **12a** | Describe all statistical methods, including those used to control for confounding. | Methods §'Multivariable Logistic Regression': full covariate set; rms package; adjustment variables listed. | **✅** |  |
| **12b** | Describe any methods used to examine subgroups and interactions. | Methods §'Interaction Analysis': formal Wald test for SUA×sex; sex-stratified RCS curves (Figure 3); Table 3. | **✅** |  |
| **12c** | Explain how missing data were addressed. | Supplementary Methods S3: SUA complete for all 21,459 participants; all covariates <5% missing; complete-case analysis used. | **✅** | Added in SupplementaryMaterial. |
| **12d** | If applicable, explain how loss to follow-up was addressed. | N/A — retrospective registry cohort; outcomes ascertained from registry records at 1 year; no active follow-up loss reported in parent study. | **N/A** | Secondary analysis; follow-up completeness governed by parent study. |
| **12e** | Describe any sensitivity analyses. | Methods §'Mendelian Randomization' (4 MR methods); Results §'Mendelian Randomization'; NIHSS≤15 sensitivity (Table S5); sex-stratified analysis as sensitivity check. | **✅** |  |
| **RESULTS** | | | | |
| **13a** | Report numbers of individuals at each stage of study — e.g. numbers potentially eligible, examined, confirmed eligible, included in the study, completing follow-up, and analysed. | Figure 1 (flowchart): screening→exclusions→final cohort (n=21,459); Results §'Baseline Characteristics'. | **✅** |  |
| **13b** | Give reasons for non-participation at each stage. | Figure 1 flowchart legend: exclusion reasons listed (prior stroke/TIA, TBI, vascular malformations, missing data). | **✅** |  |
| **13c** | Consider use of a flow diagram. | Figure 1: cohort flow diagram provided. | **✅** |  |
| **14a** | Give characteristics of study participants (e.g. demographic, clinical, social) and information on exposures and potential confounders. | Table 1: baseline characteristics by PSE status (age, sex, NIHSS, cortical involvement, SUA, urea, creatinine). | **✅** |  |
| **14b** | Indicate number of participants with missing data for each variable of interest. | Supplementary Methods S3: SUA complete; other covariates <5% missing (no individual variable counts available from de-identified public dataset). | **✅** | Limitation acknowledged; exact per-variable counts not available from de-identified Dryad dataset. |
| **14c** | Summarise follow-up time (e.g., average and total amount). | Methods §'Outcome Definition': 1-year follow-up; retrospective registry; all patients had ≥1-year record or outcome event documented. | **✅** |  |
| **15** | Report numbers of outcome events or summary measures over time. | Results §'Baseline Characteristics': 936 PSE events (4.36%); Table 3: events per sex×tertile cell. | **✅** |  |
| **16a** | Give unadjusted and, if applicable, confounder-adjusted estimates and their precision (e.g., 95% CI). Make clear which confounders were adjusted for and why they were included. | Results §'Multivariable Logistic Regression': adjusted ORs with 95% CI; Table 3: adjusted ORs by sex tertile; covariate rationale in Methods. | **✅** |  |
| **16b** | Report category boundaries when continuous variables were categorized. | Table 3 header and Methods §'Interaction Analysis': SUA tertile cut-points defined by overall cohort distribution; exact boundaries in Table S8. | **✅** |  |
| **16c** | If relevant, consider translating estimates of relative risk into absolute risk for a meaningful time period. | Table 3: incidence rates (%) reported per sex×tertile cell alongside adjusted ORs. | **✅** |  |
| **17** | Report other analyses done — e.g. analyses of subgroups and interactions, and sensitivity analyses. | Results §'Sex Interaction': formal interaction P=0.0002; sex-stratified curves Figure 3; MR sensitivity analyses Table S7; NIHSS≤15 Table S5. | **✅** |  |
| **DISCUSSION** | | | | |
| **18** | Summarise key results with reference to study objectives. | Discussion §'Principal Findings': null MR result, nonlinear observational association, sex divergence summarised with reference to two stated objectives. | **✅** |  |
| **19** | Discuss limitations of the study, taking into account sources of potential bias or imprecision. Discuss both direction and magnitude of any potential bias. | Discussion §'Strengths and Limitations': secondary analysis design, single-country cohort, unmeasured confounders, SUA timing, GWAS proxy phenotype — direction of bias discussed. | **✅** |  |
| **20** | Give a cautious overall interpretation of results considering objectives, limitations, multiplicity of analyses, and results from similar studies. | Discussion §'Clinical Implications' and §'Conclusion': interpretation caveated by null MR; consistent with Wang & Chen 2025 divergence addressed in §'Comparison with Previous Studies'. | **✅** |  |
| **21** | Discuss the generalisability (external validity) of the study results. | Discussion §'Strengths and Limitations': multicenter Chinese cohort; generalisability to other ethnic groups noted as limitation. | **✅** |  |
| **OTHER INFORMATION** | | | | |
| **22** | Give the source of funding and the role of the funders for the present study and, if applicable, for the original study on which the present article is based. | Funding Statement: no external funding for secondary analysis; parent study funding (CDJYGRH-ZD06; CNSR-III) declared with statement that funders had no role in study conduct. | **✅** |  |
| Legend: ✅ = Item addressed in manuscript or supplementary material \| N/A = Not applicable to this study design | | | | |

*All 22 STROBE items addressed. Items 10 and 12c added in Supplementary Methods S3 (SupplementaryMaterial.docx).*
