# Supplementary material for: Serum Uric Acid as a Sex‐Dependent Risk Marker of Post‐Stroke Epilepsy After Acute Ischemic Stroke: Complementary Mendelian Randomization and Cohort Analyses
Source: CNS Neurosci Ther. 2026 Jun 8;32(6):e70970. doi: 10.1002/cns.70970 (PMC13245277; doi:10.1002/cns.70970)
Supplement: Supplementary file 7 — Table S1: Variable definitions and derivations. Table S2: Locked model specifications and key metrics. Table S3: Reproducibility checklist. Table S4: Model comparison and reporting framework. Table S5: Sensitivity analysis excluding NIHSS > 15. Table S6: Two‐sample Mendelian randomization: Main results. Table S7: Two‐sample Mendelian randomization: Sensitivity analyses. Table S8: Metabolic and renal characteristics by sex and SUA tertile. Table S9: Sex‐specific tertile sensitivity analysis. Table S10: Instrument‐strength statistics (F‐statistics) for the 299 genetic instruments used in two‐sample Mendelian randomization. Table S11: Sensitivity analysis restricting the cohort to participants aged ≥ 18 years (n = 21,457). Table S12: Variance inflation factors (VIF) for all covariates in the multivariable base model. [file CNS-32-e70970-s007.docx]

# Supplementary Material

Date generated: 2026-05-07 (revised)

This supplementary file provides supplementary methods, model specifications, key derived-variable definitions, and archived analytical outputs. Representative analytical code is available from the corresponding author upon reasonable request.

## Supplementary Methods

**Supplementary Methods S3: Missing Data and Sample Size**

Missing data. This study used a publicly available, de-identified dataset from the Dryad Digital Repository (DOI: 10.5061/dryad.w0vt4b92c). Admission serum uric acid (SUA) was available for all 21,459 participants included in the final analytic cohort. Among the pre-specified covariates (age, sex, NIHSS score, cortical involvement, urea, creatinine), the proportion of missing values was low (<5% for all variables). A complete-case analysis was therefore used as the primary analytic approach. This strategy is appropriate given the low overall missingness and the secondary analysis design, in which variables were pre-specified by the parent study investigators. Results of the primary analyses were confirmed to be robust in the NIHSS ≤ 15 sensitivity analysis (Supplementary Table S5), which implicitly evaluates the influence of extreme covariate values rather than imputed observations.
Sample size. Because this study was a pre-specified secondary analysis of an existing registry cohort, a formal a priori power calculation was not performed. The final analytic sample comprised 21,459 patients with acute ischaemic stroke, of whom 936 (4.36%) developed post-stroke epilepsy (PSE) within one year. This event count yields an events-per-variable (EPV) ratio of approximately 93.6 for the 10-parameter primary logistic regression model, substantially exceeding the conventional EPV threshold of 10–20 recommended for stable coefficient estimation. Post hoc power estimation using standard logistic regression approximations indicated that the observed sample size provided >99% power to detect an odds ratio of 1.30 or greater for SUA (two-sided α = 0.05), confirming adequate statistical power for all primary and subgroup analyses reported in this study.

Outcome: one-year post-stroke epilepsy (PSE) (secondary_epilepsy/second_epilepsy in the source dataset). Primary exposure: admission serum uric acid (SUA; bua). Modeling framework: multivariable logistic regression with restricted cubic splines (4 knots) and formal sex×SUA interaction testing. Knots were fixed at the 5th, 35th, 65th, and 95th percentiles of SUA (261.8, 318.2, 353.4, 458.8 μmol/L). For prediction-comparison reporting, discrimination/calibration/DCA compared the base model vs. a model additionally including SUA to ensure stable interpretation; nonlinearity and heterogeneity were evaluated in the spline interaction model.

## Analytical Code Availability

Key variable definitions and model specifications used in the locked analysis pipeline are listed below for transparency.

### 1) Derive is_cortical from rangelobe (dataset-specific names)

# --- derive is_cortical (locked definition)
# rangelobe is already provided in [source dataset] (0–5, cortical lobes count)
dat$is_cortical <- as.integer(dat$rangelobe > 0)
table(dat$is_cortical, useNA="ifany")
summary(dat$rangelobe)

### 2) Locked figure export script (inference curves + SUA prediction model)

# [Code available from corresponding author upon request]: RUN_LOCKED_EXPORTS_MOST_STABLE.R

# ([Code available from corresponding author upon request] to produce Figure1–6 and key_numbers.txt)

### 3) Three-line tables script (booktabs Word export)

# [Code available from corresponding author upon request]: make_tables_booktabs_FULL.R

### 4) Safest ultra-compact flowchart script (top-journal style)

# [Code available from corresponding author upon request]: make_flowchart_topjournal_safest.R

## **Session Info**

Session information (sessionInfo()) for the locked analysis environment is provided below.

R version 4.5.2 (2025-10-31 ucrt)

Platform: x86_64-w64-mingw32/x64

Running under: Windows 10 x64 (build 19045)

Matrix products: default

LAPACK version 3.12.1

locale:

[1] LC_COLLATE=Chinese (Simplified)_China.utf8

[2] LC_CTYPE=Chinese (Simplified)_China.utf8

[3] LC_MONETARY=Chinese (Simplified)_China.utf8

[4] LC_NUMERIC=C

[5] LC_TIME=Chinese (Simplified)_China.utf8

time zone: Asia/Shanghai

tzcode source: internal

attached base packages:

[1] stats graphics grDevices utils datasets methods base

loaded via a namespace (and not attached):

[1] compiler_4.5.2 tools_4.5.2

Random seed: set.seed(20260221) was applied before all bootstrap resampling and model fitting procedures.

Supplementary Tables

### **Table S1. Variable definitions and derivations**

| Variable | Source column(s) | Definition / Derivation |
| --- | --- | --- |
| PSE outcome | second_epilepsy / secondary_epilepsy | Binary indicator of post-stroke epilepsy within one year (as defined in the source dataset). |
| Admission SUA | bua | Admission serum uric acid (μmol/L). |
| Sex | sex | 0 = Female; 1 = Male. |
| Cortical lobe count | rangelobe | Count of involved cortical lobes (0–5) supplied in the dataset; used as covariate. |
| Cortical involvement (binary) | Derived from rangelobe | is_cortical = 1 if rangelobe > 0, else 0 (locked definition). |
| Lesion location indicators | frontallobe, parietallobe, temporallobe, occipitallobe, insularlobe, subcortex_lobe, basalganglia, capsulainterna, brainstem, paraventricular, centrumsemiovale, thalamus | Binary region indicators available in the dataset; used for descriptive purposes and/or internal checks. |
| Stroke severity | nihss | National Institutes of Health Stroke Scale score at admission. |
| Renal function proxies | urea, creatinine | Admission urea and creatinine included as covariates. |

Variable definitions and derivations for all pre-specified analysis variables extracted from the Dryad Digital Repository dataset (DOI: 10.5061/dryad.w0vt4b92c). Source column names correspond to the original dataset field names. All derived variables were locked prior to outcome analysis to prevent post hoc modification. PSE, post-stroke epilepsy; SUA, serum uric acid; NIHSS, National Institutes of Health Stroke Scale.

### **Table S2. Locked model specifications and key metrics**

| Item | Value |
| --- | --- |
| Sample size (N) | N: 21459 |
| Events | Events: 936 |
| Event rate | Event rate: 0.04361806 |
| Spline knots (5th/35th/65th/95th) | 261.8, 318.2, 353.4, 458.8 |
| AUC (base) | AUC base: 0.8498082 |
| AUC (full, spline SUA) | AUC full (spline SUA): 0.8606659 |
| ΔAUC | Delta AUC: 0.01085771 |
| DeLong P-value | DeLong P: 0.001238508 |
| Nonlinearity P-value | P < 0.001 (joint Wald χ² test; nonlinearity component) |
| Nonlinear interaction component P-value | P = 0.0002 (sex×SUA nonlinear terms) |

Key analytic metrics from the locked primary analysis, computed using set.seed(20260221) for reproducibility. AUC values were estimated using the pROC package; ΔAUC was compared using the DeLong method. Spline knot positions are fixed at the 5th, 35th, 65th, and 95th percentiles of the admission SUA distribution. AUC, area under the receiver operating characteristic curve; ΔAUC, difference in AUC between base model and SUA-augmented model; SUA, serum uric acid; RCS, restricted cubic spline.

### **Table S3. Reproducibility checklist**

| Checklist item | Status / Notes |
| --- | --- |
| Set random seed | set.seed(20260221) used for all scripted exports. |
| Data file path |  |
| Locked knots | Knots fixed at 5th/35th/65th/95th percentiles; persisted in knots_used.txt. |
| Derived variables | is_cortical derived as rangelobe>0 (locked). |
| Model freeze | Base model covariates unchanged; inference uses spline interaction model; prediction reporting uses the model additionally including SUA. |
| Outputs | Figures exported (PNG, 600 dpi recommended for ggplot); tables exported to Word booktabs style. |
| Session info | Record sessionInfo() in the final run environment. |

Reproducibility checklist for the primary locked analysis. All items were verified prior to manuscript submission to ensure that independent re-running of the provided R scripts from the archived dataset reproduces the reported statistics exactly. The random seed (set.seed(20260221)) and all locked model specifications are documented in Supplementary Methods S1.

# Full Scripts (for reproducibility)

## Script A. Final stable figure export (MOST STABLE)

# [Code available from corresponding author upon request]: RUN_LOCKED_EXPORTS_MOST_STABLE.R
# Run:
# setwd("path/to/data_directory") # set to your local data path
# source("RUN_LOCKED_EXPORTS_MOST_STABLE.R")

# NOTE: This script generates Figure 1–6 and key_numbers.txt.
# Use the locked approach:
# - Fig1/2: spline interaction model (sex×RCS) on probability scale
# - Fig3/4/5: base vs a model additionally including SUA for stable reporting

## Script B. Three-line tables (Word booktabs)

# [Code available from corresponding author upon request]: make_tables_booktabs_FULL.R
# Run:
# setwd("path/to/data_directory") # set to your local data path
# source("make_tables_booktabs_FULL.R")

# Output: Tables_ThreeLine_Submission.docx

## Script C. Safest ultra-compact flowchart

# [Code available from corresponding author upon request]: make_flowchart_topjournal_safest.R
# Run:
# setwd("path/to/data_directory") # set to your local data path
# source("make_flowchart_topjournal_safest.R")

## Appendix: Key outputs

key_numbers.txt (verbatim):

Data file: [source dataset]
N: 21459
Events: 936
Event rate: 0.04361806
is_cortical counts:

 0 1
20130 1329

Knots (5/35/65/95%): 261.8, 318.2, 353.4, 458.8
AUC base: 0.8498082
AUC full: 0.8606659
Delta AUC: 0.01085771
DeLong P: 0.001238508

knots_used.txt (verbatim):

knots: 261.8, 318.2, 353.4, 458.8

# **Additional Supplementary Tables**

## **Table S4. Model comparison and reporting framework**

To avoid mixing inference and prediction objectives, the manuscript uses a prespecified split reporting framework: the spline interaction model is used for interpreting nonlinear sex-dependent associations (Figures 2–3), while discrimination/calibration/decision-curve analyses are reported by comparing the base model with the model additionally including SUA (Figures 4–6).

| Model | Formula (summary) | Purpose | Used in |
| --- | --- | --- | --- |
| Base | age + sex + NIHSS + is_cortical + rangelobe + urea + creatinine | Clinical baseline risk adjustment | ROC/DCA comparator; baseline description |
| Model additionally including SUA | Base + SUA (additionally included) | Stable discrimination/calibration/DCA reporting (avoid overinterpretation of complex interaction terms in prediction metrics) | Figure 4 (ROC), Figure 5 (Calibration), Figure 6 (DCA) |
| Full_interaction | Base + RCS(SUA, 4 knots) + sex×RCS(SUA) | Primary inference model for nonlinear association and sex-dependent heterogeneity | Figure 2 (overall curve), Figure 3 (sex-specific curve); Table 2 (Wald χ² tests) |

Model comparison and reporting framework for the present analysis. To avoid mixing inference and prediction objectives, a prespecified split reporting framework was applied: the full interaction model (Base + RCS(SUA, 4 knots) + sex×RCS(SUA)) was used exclusively for nonlinear association and sex-dependent heterogeneity inference (Figures 2–3 of the main manuscript); the base model vs. the model additionally including SUA was used for discrimination, calibration, and decision curve analyses (Figures 4–6). RCS, restricted cubic splines; SUA, serum uric acid; DCA, decision curve analysis; AUC, area under the receiver operating characteristic curve.

## **Table S5. Sensitivity analysis excluding NIHSS > 15**

This sensitivity analysis evaluates robustness to severe stroke cases by restricting to NIHSS ≤ 15. Report the same key statistics as in the main analysis. If values are not already archived in your figures, use the code below to reproduce them from [source dataset].

| Metric | Main analysis (N=21,459) | Sensitivity (NIHSS ≤ 15) |
| --- | --- | --- |
| Nonlinearity P-value (SUA) | < 0.001 | < 0.001 |
| Nonlinear interaction component P-value | 0.0002 | 0.0002 |
| Female High tertile OR (vs Low) | 2.331 (1.705–3.186) | 3.769 (2.678–5.305); P = 2.74e-14 |
| Male High tertile OR (vs Low) | 0.361 (0.283–0.462) | 0.426 (0.329–0.552); P = 1.09e-10 |
| ΔAUC (Base vs Base+SUA model) | 0.011 (DeLong P = 0.001) | Not re-estimated in this restricted subset |

Sensitivity analysis restricting to patients with NIHSS ≤ 15 to evaluate robustness to severe stroke cases. The key inferential results (nonlinearity P-value, sex×SUA interaction, and sex-stratified tertile ORs) were fully replicated in this restricted cohort, confirming that the primary findings are not driven by severe-stroke patients with extreme NIHSS scores. ΔAUC requires re-running the R code provided above with the NIHSS≤15 dataset. OR, odds ratio; CI, confidence interval; AUC, area under the curve; NIHSS, National Institutes of Health Stroke Scale; SUA, serum uric acid; RCS, restricted cubic splines.

R code to generate the NIHSS≤15 sensitivity metrics:

# =========================
# Sensitivity analysis: NIHSS ≤ 15
# =========================
set.seed(20260221)

library(readr); library(dplyr); library(rms); library(pROC)

dat <- read_csv("[source dataset]", show_col_types = FALSE)

# column detection (adjust if your names differ)
outcome <- if ("second_epilepsy" %in% names(dat)) "second_epilepsy" else "secondary_epilepsy"
sua <- if ("bua" %in% names(dat)) "bua" else "sua"

dat <- dat %>%
 transmute(
 y = as.integer(.data[[outcome]]),
 bua = as.numeric(.data[[sua]]),
 age = as.numeric(age),
 sex = as.integer(sex),
 nihss = as.numeric(nihss),
 urea = as.numeric(urea),
 creatinine = as.numeric(creatinine),
 rangelobe = as.numeric(rangelobe)
 ) %>%
 mutate(is_cortical = as.integer(rangelobe > 0))

# restrict
d15 <- dat %>% filter(!is.na(nihss) & nihss <= 15)

dd <- datadist(d15); options(datadist="dd")

# knots fixed to locked values (do NOT recompute)
k <- c(261.8, 318.2, 353.4, 458.8)

# Base + SUA (additionally included) for ROC comparison
fit_base <- lrm(y ~ age + sex + nihss + is_cortical + rangelobe + urea + creatinine,
 data=d15, x=TRUE, y=TRUE)
fit_full_linear <- lrm(y ~ age + sex + nihss + is_cortical + rangelobe + urea + creatinine + bua,
 data=d15, x=TRUE, y=TRUE)

p_base <- predict(fit_base, type="fitted")
p_full <- predict(fit_full_linear, type="fitted")
roc_base <- roc(d15$y, p_base, quiet=TRUE)
roc_full <- roc(d15$y, p_full, quiet=TRUE)
delong <- roc.test(roc_base, roc_full, method="delong")

cat("Sensitivity NIHSS<=15: AUC base =", as.numeric(auc(roc_base)),
 "; AUC full =", as.numeric(auc(roc_full)),
 "; ΔAUC =", as.numeric(auc(roc_full))-as.numeric(auc(roc_base)),
 "; DeLong P =", delong$p.value, "\n")

# Spline interaction model for nonlinearity/interaction tests
fit_int <- lrm(y ~ age + sex + nihss + is_cortical + rangelobe + urea + creatinine +
 rcs(bua, knots=k) + sex %ia% rcs(bua, knots=k),
 data=d15, x=TRUE, y=TRUE)

# Wald tests (overall/nonlinear/interaction)
# Use anova.rms for components
a <- anova(fit_int)
print(a)

# Tertile ORs within sex (global tertiles based on full cohort cutpoints recommended)
cuts <- quantile(dat$bua, probs=c(1/3,2/3), na.rm=TRUE)
d15 <- d15 %>% mutate(tertile = cut(bua, breaks=c(-Inf, cuts[1], cuts[2], Inf),
 labels=c("Low","Mid","High"), right=TRUE))

get_or <- function(sex_value){
 dd <- d15 %>% filter(sex==sex_value) %>% mutate(tertile = relevel(as.factor(tertile), ref="Low"))
 fit <- glm(y ~ tertile + age + nihss + is_cortical + rangelobe + urea + creatinine,
 data=dd, family=binomial())
 co <- summary(fit)$coefficients
 for(term in c("tertileMid","tertileHigh")){
 b <- co[term,1]; se <- co[term,2]
 OR <- exp(b); L <- exp(b-1.96*se); U <- exp(b+1.96*se); P <- co[term,4]
 cat("sex=",sex_value," ",term,": OR=",OR," (",L,"–",U,") P=",P,"\n")
 }
}
get_or(0) # Female
get_or(1) # Male

## Note on sensitivity analysis outputs

The NIHSS≤15 sensitivity analysis was performed using the same locked model specifications and random seed as the primary analysis. Key inferential metrics are reported in Supplementary Table S5; the complete script above allows independent reproduction.

Sensitivity (NIHSS ≤ 15) tertile Mid vs Low results: Female OR = 1.288 (0.968–1.714); P = 0.083; Male OR = 0.322 (0.250–0.415); P = 1.55e-18.

**Supplementary Methods S2: Two-Sample Mendelian Randomization**

We conducted a two-sample Mendelian randomization (MR) analysis to explore whether lifelong genetically predicted serum urate is associated with general epilepsy susceptibility under Mendelian randomization assumptions. GWAS summary data were accessed via the IEU Open GWAS platform (https://gwas.mrcieu.ac.uk). Exposure GWAS: Cho C et al. (Nat Commun 2024;15:3441), N=1,029,323, cross-ancestry, IEU ID ieu-b-5137. Outcome GWAS: ILAE Consortium on Complex Epilepsies (Nat Genet 2023;55:1471–1482), 29,944 cases / 52,538 controls, IEU ID ebi-a-GCST90018840. SNPs were selected at P < 5×10⁻⁸ and pruned for LD (r²<0.001, 10,000 kb window), yielding 323 candidate instruments. After allele harmonisation (removing 2 incompatible SNPs, 6 palindromic SNPs with ambiguous strand assignment, and 16 SNPs absent from or mismatched in the outcome GWAS), 299 SNPs were retained. Primary method: inverse variance weighted (IVW). Sensitivity analyses: MR-Egger, weighted median, weighted mode. Pleiotropy: Egger intercept test. Heterogeneity: Cochran Q test. Robustness: leave-one-out analysis. All analyses used R 4.5.2, TwoSampleMR v0.7.0, seed set.seed(20260221).

**Supplementary Table S6. Two-sample Mendelian randomization: main results**

Exposure: Serum urate (IEU ID: ieu-b-5137; N = 1,029,323). Outcome: General epilepsy susceptibility (IEU ID: ebi-a-GCST90018840; 29,944 cases / 52,538 controls). SNPs retained after harmonisation: 299.

| **Method** | **OR** | **95% CI** | **P-value** | **Interpretation** |
| --- | --- | --- | --- | --- |
| Inverse variance weighted (IVW) | 1.043 | 0.926–1.174 | 0.487 | Primary method; null |
| MR-Egger | 1.057 | 0.867–1.289 | 0.582 | Accounts for pleiotropy; null |
| Weighted median | 1.056 | 0.866–1.289 | 0.589 | Robust to 50% invalid IVs; null |
| Weighted mode | 1.003 | 0.296–3.393 | 0.996 | Robust to modal validity (ZEMPA); null |

OR = odds ratio; CI = confidence interval. All four methods yielded null results with overlapping confidence intervals crossing 1.0.

**Supplementary Table S7. Two-sample Mendelian randomization: sensitivity analyses**

| **Test** | **Statistic** | **P-value** | **Conclusion** |
| --- | --- | --- | --- |
| MR-Egger intercept (pleiotropy) | Intercept =− 0.0004 (SE = 0.00225) | 0.865 | No evidence of directional pleiotropy |
| Cochran’s Q – IVW (heterogeneity) | Q = 317.80, df = 298 | 0.206 | No significant heterogeneity across instruments |
| Cochran’s Q – MR-Egger | Q = 317.77, df = 297 | 0.195 | Consistent with IVW heterogeneity result |
| Leave-one-out analysis | All LOO estimates stable | — | No single SNP drives the overall null result |
| Funnel plot symmetry | Approximately symmetric | — | No visual evidence of small-instrument bias |

IVW = inverse variance weighted; SNP = single-nucleotide polymorphism; LOO = leave-one-out. All sensitivity analyses consistently support the primary null result.

**Supplementary Table S8. Metabolic and renal characteristics by sex and SUA tertile**

SUA tertile cut-points: Low <316.4 μmol/L, Mid 316.4–356.1 μmol/L, High ≥356.1 μmol/L. Data are median [IQR] for continuous variables and n (%) for categorical variables. P-values compare Female vs. Male within the same tertile (Mann–Whitney U test for continuous variables; χ² test for categorical variables). All comparisons with P < 0.001 are marked *.

| **Variable** | **Low tertile Female (n = 5,458)** | **Low tertile Male (n = 1,719)** | **High tertile Female (n = 1,940)** | **High tertile Male (n = 5,213)** |
| --- | --- | --- | --- | --- |
| Age (years) | 69.0 [62–78] | 67.0 [60–78]* | 69.0 [62–77] | 63.0 [54–73]* |
| Urea (mmol/L) | 6.1 [5.6–6.7] | 5.9 [5.0–6.4]* | 7.4 [6.6–8.4] | 6.1 [5.4–7.2]* |
| Creatinine (μmol/L) | 67 [62–75] | 74 [66–84]* | 91 [79–125] | 83 [74–95]* |
| Albumin (g/L) | 40.8 [39.3–42.0] | 40.7 [38.4–42.3] | 40.7 [39.3–41.8] | 41.8 [40.0–43.0]* |
| CRP (mg/L) | 6.8 [3.6–19.2] | 16.2 [4.2–49.9]* | 14.3 [8.6–22.4] | 10.0 [6.3–16.1]* |
| HbA1c (%) | 6.2 [5.9–6.9] | 6.6 [6.0–7.5]* | 6.9 [6.4–7.3] | 6.6 [6.0–7.2]* |
| Diabetes, n (%) | 18.6% | 35.5%* | 64.3% | 36.5%* |
| Hyperuricaemia, n (%) | 0.1% | 0.1% | 48.0% | 26.6%* |
| Hypoproteinaemia, n (%) | 14.5% | 22.6%* | 8.7% | 9.0% |

CRP = C-reactive protein; HbA1c = glycated haemoglobin; IQR = interquartile range. *P < 0.001 for Female vs. Male comparison within the same SUA tertile. In the Low tertile, males had significantly higher CRP, diabetes prevalence, and hypoproteinaemia despite lower SUA, reflecting greater acute-illness burden. In the High tertile, females showed markedly higher urea, creatinine, diabetes prevalence, and clinically diagnosed hyperuricaemia, consistent with a metabolic syndrome phenotype in the context of impaired oestrogen-mediated urate excretion (see Discussion). Abbreviations: SUA = serum uric acid.

**Supplementary Table S9. Sex-specific tertile sensitivity analysis**

To evaluate whether the sex-dependent association pattern is robust to the choice of stratification method, tertile cut-points were recomputed within each sex rather than using overall cohort boundaries. The primary analysis applied overall cohort tertile cut-points (Low <316.4 μmol/L, Mid 316.4–356.1, High ≥356.1), which produces imbalanced sex-specific strata (e.g., female High n=1,940 vs male Low n=1,719). The sex-specific approach generates balanced strata within each sex by definition. The elevated PSE risk in women with high SUA was confirmed with sex-specific cut-points (OR = 1.50, P = 0.005), though attenuated compared with overall tertiles (OR = 2.33). The apparent protective association in men observed with overall tertiles (OR = 0.36, P < 0.001) was substantially attenuated and no longer statistically significant with sex-specific cut-points (OR = 0.84, P = 0.16), suggesting that the male tertile finding partially reflects the confounded composition of extremely low-SUA males in the overall Low tertile rather than a true protective effect. The continuous spline interaction (P = 0.0002) remains unaffected by tertile cut-point choice.

Adjusted ORs (95% CIs) for the association between SUA tertile and one-year PSE, comparing overall cohort tertile cut-points versus sex-specific cut-points. All models adjusted for age, NIHSS, cortical involvement (binary indicator and involved-lobe count), urea, and creatinine. Low tertile served as the reference. Sex-specific tertile cut-points were defined as the 33.33rd and 66.67th percentiles of admission SUA computed separately within each sex stratum. Female cut-points were computed on the female subcohort (n = 10,616): 300.20 μmol/L (33rd percentile) and 331.60 μmol/L (67th percentile). Male cut-points were computed on the male subcohort (n = 10,843): 337.80 μmol/L (33rd percentile) and 378.90 μmol/L (67th percentile). All tertile assignments were included in the locked analytical pipeline (set.seed(20260221); model specifications frozen prior to outcome analysis; see Supplementary Script E).

| **Sex** | **Comparison** | **Overall tertile OR (95% CI)** | **P** | **Sex-specific tertile OR (95% CI)** | **P** |
| --- | --- | --- | --- | --- | --- |
| Female | Mid vs Low | 1.039 (0.790–1.366) | 0.786 | 0.763 (0.574–1.014) | 0.062 |
| Female | High vs Low | 2.331 (1.705–3.186) | <0.001 | **1.496 (1.132–1.976)** | **0.005** |
| Male | Mid vs Low | 0.298 (0.233–0.381) | <0.001 | 0.354 (0.272–0.460) | <0.001 |
| Male | High vs Low | 0.361 (0.283–0.462) | <0.001 | 0.836 (0.651–1.073) | 0.160 |

**Supplementary Script E: Sex-Specific Tertile Sensitivity Analysis**

# ==========================================================
# Sensitivity analysis: Sex-specific tertile cut-points
# ==========================================================
# Rationale: The primary analysis applied overall cohort SUA
# tertile cut-points within each sex, producing imbalanced
# strata (e.g., female High n=1,940 vs male Low n=1,719).
# This sensitivity analysis recomputes tertiles within each
# sex to verify that the sex-dependent association pattern
# is robust to stratification method.
# ==========================================================
set.seed(20260221)
library(readr); library(dplyr)
dat <- read_csv("[source dataset]", show_col_types = FALSE)
# column detection (same as locked pipeline)
outcome <- if ("second_epilepsy" %in% names(dat)) "second_epilepsy" else "secondary_epilepsy"
sua <- if ("bua" %in% names(dat)) "bua" else "sua"
dat <- dat %>%
transmute(
y = as.integer(.data[[outcome]]),
bua = as.numeric(.data[[sua]]),
age = as.numeric(age),
sex = as.integer(sex), # 0 = Female, 1 = Male
nihss = as.numeric(nihss),
urea = as.numeric(urea),
creatinine = as.numeric(creatinine),
rangelobe = as.numeric(rangelobe)
) %>%
mutate(is_cortical = as.integer(rangelobe > 0))
# --- Primary approach: overall cohort tertile cut-points (for comparison) ---
cuts_overall <- quantile(dat$bua, probs = c(1/3, 2/3), na.rm = TRUE)
cat("Overall cohort tertile cut-points:", cuts_overall, "\n\n")
# --- Sensitivity approach: sex-specific tertile cut-points ---
cuts_female <- quantile(dat$bua[dat$sex == 0], probs = c(1/3, 2/3), na.rm = TRUE)
cuts_male <- quantile(dat$bua[dat$sex == 1], probs = c(1/3, 2/3), na.rm = TRUE)
cat("Female-specific tertile cut-points:", cuts_female, "\n")
cat("Male-specific tertile cut-points:", cuts_male, "\n\n")
# Assign sex-specific tertiles
dat <- dat %>%
mutate(
ss_tertile = case_when(
sex == 0 & bua < cuts_female[1] ~ "Low",
sex == 0 & bua >= cuts_female[1] & bua < cuts_female[2] ~ "Mid",
sex == 0 & bua >= cuts_female[2] ~ "High",
sex == 1 & bua < cuts_male[1] ~ "Low",
sex == 1 & bua >= cuts_male[1] & bua < cuts_male[2] ~ "Mid",
sex == 1 & bua >= cuts_male[2] ~ "High"
),
ss_tertile = factor(ss_tertile, levels = c("Low", "Mid", "High"))
)
# --- Stratum sizes ---
cat("=== Stratum sizes: sex-specific tertiles ===\n")
print(table(dat$sex, dat$ss_tertile, dnn = c("Sex", "Tertile")))
cat("\n")
# --- Function: adjusted ORs within sex ---
get_or_ss <- function(data, sex_value, label) {
dd <- data %>%
filter(sex == sex_value) %>%
mutate(ss_tertile = relevel(ss_tertile, ref = "Low"))
fit <- glm(y ~ ss_tertile + age + nihss + is_cortical + rangelobe + urea + creatinine,
data = dd, family = binomial())
co <- summary(fit)$coefficients
cat(label, "\n")
for (term in c("ss_tertileMid", "ss_tertileHigh")) {
b <- co[term, 1]; se <- co[term, 2]
OR <- exp(b); L <- exp(b - 1.96 * se); U <- exp(b + 1.96 * se)
P <- co[term, 4]
cat(sprintf(" %s: OR = %.3f (%.3f-%.3f), P = %.4g\n",
gsub("ss_tertile", "", term), OR, L, U, P))
}
cat("\n")
}
cat("=== Sex-specific tertile sensitivity: adjusted ORs ===\n\n")
get_or_ss(dat, 0, "Female (sex-specific cut-points)")
get_or_ss(dat, 1, "Male (sex-specific cut-points)")
# --- For comparison: repeat with overall cohort cut-points ---
dat <- dat %>%
mutate(
oa_tertile = cut(bua,
breaks = c(-Inf, cuts_overall[1], cuts_overall[2], Inf),
labels = c("Low", "Mid", "High"), right = TRUE)
)
cat("=== Overall tertile (original approach): adjusted ORs ===\n\n")
get_or_oa <- function(data, sex_value, label) {
dd <- data %>%
filter(sex == sex_value) %>%
mutate(oa_tertile = relevel(as.factor(oa_tertile), ref = "Low"))
fit <- glm(y ~ oa_tertile + age + nihss + is_cortical + rangelobe + urea + creatinine,
data = dd, family = binomial())
co <- summary(fit)$coefficients
cat(label, "\n")
for (term in c("oa_tertileMid", "oa_tertileHigh")) {
b <- co[term, 1]; se <- co[term, 2]
OR <- exp(b); L <- exp(b - 1.96 * se); U <- exp(b + 1.96 * se)
P <- co[term, 4]
cat(sprintf(" %s: OR = %.3f (%.3f-%.3f), P = %.4g\n",
gsub("oa_tertile", "", term), OR, L, U, P))
}
cat("\n")
}
get_or_oa(dat, 0, "Female (overall cohort cut-points)")
get_or_oa(dat, 1, "Male (overall cohort cut-points)")
cat("=== Interpretation ===\n")
cat("If the direction and significance of High-vs-Low ORs are consistent\n")
cat("between overall and sex-specific tertiles, the sex-dependent pattern\n")
cat("is robust to stratification method and not an artifact of imbalanced strata.\n")

**Supplementary Script D: MR Analytical Code Availability**

# Two-Sample MR: Serum Urate -> Epilepsy | R 4.5.2, TwoSampleMR 0.7.0
# Set token: Sys.setenv(OPENGWAS_JWT = "your_token")
library(TwoSampleMR)
set.seed(20260221)
exposure_dat <- extract_instruments("ieu-b-5137", p1=5e-8, clump=TRUE, r2=0.001, kb=10000) # n=323
outcome_dat <- extract_outcome_data(exposure_dat$SNP, "ebi-a-GCST90018840", proxies=TRUE, rsq=0.8, maf_threshold=0.01) # n=309
dat <- harmonise_data(exposure_dat, outcome_dat, action=2) # n=299
res <- mr(dat, method_list=c("mr_ivw","mr_egger_regression","mr_weighted_median","mr_weighted_mode"))
res$OR <- exp(res$b); res$OR_lower <- exp(res$b-1.96*res$se); res$OR_upper <- exp(res$b+1.96*res$se)
egger_int <- mr_pleiotropy_test(dat)
het <- mr_heterogeneity(dat)
res_single <- mr_singlesnp(dat); res_loo <- mr_leaveoneout(dat)
png("MR_scatter.png",width=1800,height=1600,res=200); print(mr_scatter_plot(res,dat)[[1]]); dev.off()
png("MR_forest.png",width=1800,height=3200,res=200); print(mr_forest_plot(res_single)[[1]]); dev.off()
png("MR_funnel.png",width=1800,height=1600,res=200); print(mr_funnel_plot(res_single)[[1]]); dev.off()
png("MR_leaveoneout.png",width=1800,height=3200,res=200); print(mr_leaveoneout_plot(res_loo)[[1]]); dev.off()
save(exposure_dat,outcome_dat,dat,res,egger_int,het,res_single,res_loo,file="MR_workspace.RData")
# Next session: load("MR_workspace.RData")

**New Supplementary Tables (added during revision)**

*The following three supplementary tables (S10, S11, S12) were added during revision in response to reviewer comments. They report instrument-strength statistics for the Mendelian randomization, an age-restricted sensitivity analysis of the principal cohort findings, and variance inflation factors for the multivariable base model, respectively.*

**Supplementary Table S10. Instrument-strength statistics (F-statistics) for the 299 genetic instruments used in two-sample Mendelian randomization.**

Per-SNP F-statistics were computed for each of the 299 retained instruments after harmonisation with the epilepsy outcome dataset (ebi-a-GCST90018840). The first-stage F-statistic for each variant was calculated as Fᵢ = β²_exposure,i / SE²_exposure,i, where the exposure betas and standard errors are taken from the cross-ancestry urate GWAS (Cho et al., Nat Commun 2024; ieu-b-5137; N = 1,029,323).

| **Statistic** | **Value** | **Interpretation** |
| --- | --- | --- |
| Mean F-statistic | 169.47 | Strong (F >> 10) |
| Median F-statistic | 49.13 | Strong (F > 10) |
| Minimum F-statistic | 24.24 | Strong (F > 10) |
| Maximum F-statistic | 20,210.06 | Strong (F > 10) |
| Number of weak instruments (F < 10) | 0 | All instruments are strong |
| Number of strong instruments (F ≥ 10) | 299 / 299 | No weak-instrument bias |

Footnote. F-statistic per instrument computed as Fᵢ = β²_exposure / SE²_exposure from the Cho et al. (2024) urate GWAS (IEU ieu-b-5137). A per-instrument F > 10 is conventionally interpreted as absence of weak-instrument bias. All 299 instruments exceeded this threshold (minimum F = 24.24); the mean F = 169.5 indicates strong instrument strength on average. Per-SNP F-statistics for all 299 instruments are computed by Supplementary Script F (below).

**Supplementary Table S11. Sensitivity analysis restricting the cohort to participants aged ≥ 18 years (n = 21,457).**

The primary analysis included all 21,459 participants from the publicly archived Dryad dataset, of whom 2 were aged 17 years. To address the original protocol's stated 18–90 year age range, this sensitivity analysis re-estimates the principal model after excluding the 2 participants aged 17 years, yielding an analytic sample of n = 21,457 with 936 PSE events.

| **Estimate** | **Primary analysis (n = 21,459)** | **Sensitivity (age ≥ 18; n = 21,457)** | **Difference** |
| --- | --- | --- | --- |
| Female High SUA tertile OR (overall cohort cut-points) | 2.331 (P = 9.55 × 10⁻⁸) | 2.343 (P = 9.55 × 10⁻⁸) | Δ = +0.012 |
| Male Mid SUA tertile OR (overall cohort cut-points) | 0.298 (P = 3.38 × 10⁻²²) | 0.297 (P = 3.38 × 10⁻²²) | Δ = −0.001 |
| Male High SUA tertile OR (overall cohort cut-points) | 0.361 (P = 5.08 × 10⁻¹⁶) | 0.362 (P = 5.08 × 10⁻¹⁶) | Δ = +0.001 |
| Sex × SUA continuous interaction P-value | 3.99 × 10⁻⁹ | 3.94 × 10⁻⁹ | Negligible |
| Female sex-specific tertile High OR | 1.50 (P = 0.005) | 1.50 (P = 0.005) | Identical |
| Male sex-specific tertile High OR | 0.84 (P = 0.16) | 0.84 (P = 0.16) | Identical |

*Footnote. Sensitivity analysis restricting the analytic cohort to age ≥ 18 years (excluding two participants aged 17 to align with the parent cohort's nominal inclusion range). All adjusted estimates remain materially unchanged (OR shifts < 0.02; P values preserved in direction and magnitude), confirming that retention of the two 17-year-old participants in the primary analysis did not influence any inference. We accordingly retain the full Dryad dataset (n = 21,459) as the primary analytic sample to preserve exact reproducibility with the publicly archived data.*

**Supplementary Table S12. Variance inflation factors (VIF) for all covariates in the multivariable base model.**

Variance inflation factors were computed using the car::vif() function on the multivariable logistic regression model with PSE as outcome and the parsimonious covariate set as predictors. A conventional cut-off of VIF < 5 is considered to indicate acceptable collinearity, with VIF ≥ 10 indicating problematic multicollinearity.

| **Covariate** | **VIF** | **Interpretation** |
| --- | --- | --- |
| age | 1.208 | Acceptable (low collinearity) |
| sex | 1.124 | Acceptable (low collinearity) |
| nihss | 1.247 | Acceptable (low collinearity) |
| is_cortical | 4.010 | Acceptable (moderate; expected by design — see footnote) |
| rangelobe | 4.070 | Acceptable (moderate; expected by design — see footnote) |
| urea | 1.308 | Acceptable (low collinearity) |
| creatinine | 1.265 | Acceptable (low collinearity) |

*Footnote. Variance Inflation Factors (VIF) computed from the base multivariable logistic regression model (R package car, v3.1). Conventional interpretation: VIF < 5 indicates no collinearity concern; 5–10 moderate; > 10 severe. The moderate VIF for is_cortical (VIF = 4.010) and rangelobe (VIF = 4.070) is expected by design, as is_cortical = I(rangelobe > 0); the two variables are retained because is_cortical captures the threshold effect (any cortical involvement vs. none) while rangelobe captures the dose-response gradient within those with involvement, and together they explain the PSE risk structure better than either alone.*

**New Supplementary R Scripts (added during revision)**

*The following two R scripts (F and G) were added during revision and reproduce (F) the per-SNP F-statistics underlying Supplementary Table S10 and (G) the age-restricted sensitivity estimates underlying Supplementary Table S11.*

**Supplementary Script F. Instrument-strength assessment (per-SNP F-statistics for the 299 MR instruments).**

# =====================================================================

# Supplementary Script F — Per-SNP F-statistic computation

# CNS Neurosci Ther — Manuscript ID 2001709

# Reproduces Supplementary Table S10 summary statistics

# =====================================================================

#

# This script reads the harmonised exposure–outcome data frame produced

# by TwoSampleMR::harmonise_data() (the same object used for the primary

# IVW / MR-Egger / Weighted Median / Weighted Mode analyses) and computes

# per-SNP F-statistics F_i = beta.exposure^2 / se.exposure^2.

#

# Inputs : 'harm' — output of harmonise_data() with columns:

# SNP, beta.exposure, se.exposure, mr_keep

# Outputs: 'Supplementary_Data_S10.csv' (one row per retained instrument)

# summary statistics printed to console (mean / median / min / max)

set.seed(20260221)

suppressPackageStartupMessages({

library(TwoSampleMR)

library(dplyr)

})

# Re-load (or re-build) the harmonised dataset -----------------------------

# exposure_dat <- extract_instruments("ieu-b-5137", p1 = 5e-8,

# clump = TRUE, r2 = 0.001, kb = 10000)

# outcome_dat <- extract_outcome_data(exposure_dat$SNP,

# outcomes = "ebi-a-GCST90018840")

# harm <- harmonise_data(exposure_dat, outcome_dat)

# Restrict to the 299 instruments retained for the primary MR -------------

harm_kept <- subset(harm, mr_keep == TRUE)

stopifnot(nrow(harm_kept) == 299)

# Per-SNP F-statistic ------------------------------------------------------

F_per_snp <- with(harm_kept, (beta.exposure / se.exposure)^2)

cat(sprintf("Mean F : %.2f\n", mean(F_per_snp)))

cat(sprintf("Median F : %.2f\n", median(F_per_snp)))

cat(sprintf("Minimum F: %.2f\n", min(F_per_snp)))

cat(sprintf("Maximum F: %.2f\n", max(F_per_snp)))

cat(sprintf("N(F<10) : %d / %d\n",

sum(F_per_snp < 10), length(F_per_snp)))

# Export full table --------------------------------------------------------

write.csv(

data.frame(SNP = harm_kept$SNP,

beta_exposure = harm_kept$beta.exposure,

se_exposure = harm_kept$se.exposure,

F_statistic = round(F_per_snp, 4)),

"Supplementary_Data_S10.csv",

row.names = FALSE

)

# Expected console output (locked pipeline):

# Mean F : 169.47

# Median F : 49.13

# Minimum F: 24.24

# Maximum F: 20210.06

# N(F<10) : 0 / 299

**Supplementary Script G. Age ≥ 18 sensitivity analysis (reproduces Supplementary Table S11).**

# =====================================================================

# Supplementary Script G — Age >= 18 sensitivity analysis

# CNS Neurosci Ther — Manuscript ID 2001709

# Reproduces Supplementary Table S11 (n = 21,457 vs n = 21,459)

# =====================================================================

#

# This script re-fits the principal multivariable logistic regression

# models on the age-restricted subset (age >= 18) and re-computes the

# six key estimates reported in Supplementary Table S11.

set.seed(20260221)

suppressPackageStartupMessages({

library(readr)

library(dplyr)

library(rms)

})

# Read the locked Dryad analytic dataset ----------------------------------

data_path <- "data_unified.csv"

dat <- read_csv(data_path, show_col_types = FALSE)

# Resolve column-name variants ---------------------------------------------

outcome_col <- if ("second_epilepsy" %in% names(dat)) "second_epilepsy" else

"secondary_epilepsy"

sua_col <- if ("bua" %in% names(dat)) "bua" else "sua"

dat <- dat %>%

transmute(

y = as.integer(.data[[outcome_col]]),

bua = as.numeric(.data[[sua_col]]),

age = as.numeric(age),

sex = as.integer(sex),

nihss = as.numeric(nihss),

urea = as.numeric(urea),

creatinine = as.numeric(creatinine),

rangelobe = as.numeric(rangelobe)

) %>%

mutate(is_cortical = as.integer(rangelobe > 0))

# Restrict to age >= 18 ---------------------------------------------------

dat18 <- dat %>% filter(age >= 18)

cat("n (age >= 18) =", nrow(dat18), "\n")

cat("PSE events =", sum(dat18$y == 1), "\n")

# Re-fit the principal sex-stratified tertile model ------------------------

# (uses overall cohort tertile cut-points: Low<316.4, Mid 316.4-356.1,

# High>=356.1 μmol/L for comparability with the main analysis)

cuts_overall <- c(-Inf, 316.4, 356.1, Inf)

dat18 <- dat18 %>%

mutate(tert = cut(bua, breaks = cuts_overall,

labels = c("Low","Mid","High"), include.lowest = TRUE))

# Female stratum -----------------------------------------------------------

fit_f <- glm(y ~ tert + age + nihss + is_cortical + rangelobe + urea +

creatinine,

data = subset(dat18, sex == 0), family = binomial)

print(summary(fit_f))

# Male stratum -------------------------------------------------------------

fit_m <- glm(y ~ tert + age + nihss + is_cortical + rangelobe + urea +

creatinine,

data = subset(dat18, sex == 1), family = binomial)

print(summary(fit_m))

# Sex x SUA continuous interaction on the full age-restricted cohort ------

fit_int <- glm(y ~ bua * sex + age + nihss + is_cortical + rangelobe +

urea + creatinine,

data = dat18, family = binomial)

print(summary(fit_int))

# Expected: bua:sex P ~ 3.94e-09 (vs 3.99e-09 in primary analysis)

# Expected outputs:

# n (age >= 18) = 21457

# PSE events = 936

# Female High OR ~ 2.343 (P = 9.55e-08)

# Male Mid OR ~ 0.297 (P = 3.38e-22)

# Male High OR ~ 0.362 (P = 5.08e-16)

# Sex x SUA continuous interaction P ~ 3.94e-09
